# Signaling pathways involved in ischemic stroke: molecular mechanisms and therapeutic interventions

**DOI:** 10.1038/s41392-022-01064-1

**Published:** 2022-07-06

**Authors:** Chuan Qin, Sheng Yang, Yun-Hui Chu, Hang Zhang, Xiao-Wei Pang, Lian Chen, Luo-Qi Zhou, Man Chen, Dai-Shi Tian, Wei Wang

**Affiliations:** Department of Neurology, Tongji Hospital, Tongji Medical College, Huazhong University of Science and Technology, Wuhan, 430030 China

**Keywords:** Molecular neuroscience, Neurological disorders

## Abstract

Ischemic stroke is caused primarily by an interruption in cerebral blood flow, which induces severe neural injuries, and is one of the leading causes of death and disability worldwide. Thus, it is of great necessity to further detailly elucidate the mechanisms of ischemic stroke and find out new therapies against the disease. In recent years, efforts have been made to understand the pathophysiology of ischemic stroke, including cellular excitotoxicity, oxidative stress, cell death processes, and neuroinflammation. In the meantime, a plethora of signaling pathways, either detrimental or neuroprotective, are also highly involved in the forementioned pathophysiology. These pathways are closely intertwined and form a complex signaling network. Also, these signaling pathways reveal therapeutic potential, as targeting these signaling pathways could possibly serve as therapeutic approaches against ischemic stroke. In this review, we describe the signaling pathways involved in ischemic stroke and categorize them based on the pathophysiological processes they participate in. Therapeutic approaches targeting these signaling pathways, which are associated with the pathophysiology mentioned above, are also discussed. Meanwhile, clinical trials regarding ischemic stroke, which potentially target the pathophysiology and the signaling pathways involved, are summarized in details. Conclusively, this review elucidated potential molecular mechanisms and related signaling pathways underlying ischemic stroke, and summarize the therapeutic approaches targeted various pathophysiology, with particular reference to clinical trials and future prospects for treating ischemic stroke.

## Introduction

### Epidemiology, diagnosis, and treatment for ischemic stroke

Ischemic stroke is caused by an interruption in cerebral blood flow, induced by thrombosis or embolism. It represents the second leading cause of deaths worldwide, with 5.9 million deaths and 102 million disability-adjusted life years lost.^1,2^ Several risk factors have been implicated in the pathogenesis of ischemic stroke, including diabetes, cigarette smoking, hyperlipidemia, and hypertension.^3^ Based on the etiology, the cause of ischemic stroke can be traced to embolism from the heart, artery-to-artery embolism, and in situ small vessel disease.^2,4^ Typically, stroke symptoms include sudden unilateral weakness, numbness, diplopia, slurred speech, ataxia, and non-orthostatic vertigo.^5^ Various efforts have been made to improve outcome after stroke onset. Immediate clinical interventions, such as intravenous thrombolytic treatment and mechanical thrombectomy, contribute to the recanalization of cerebral blood vessels.^5^ While antithrombotic therapies, including antiplatelet or anticoagulant agents, are recommended for nearly all patients with no contraindication,^3^ pharmacological approaches against ischemic stroke remain limited, suggesting the need for new treatments.

### Morphological changes in ischemic stroke

In the pathogenesis of ischemic stroke, various types of cells in the central nervous system experience different morphological alterations facing ischemic damages. In the ischemic core, neurons undergo morphological changes where the cell bodies and axons disappear.^6,7^ Swelling of the cytoplasm and nucleolus disappearance are often seen in neurons as well as glial cells. While in the penumbra, neurons, which are referred to as ‘ischemic neurons’ and relatively viable, usually experience several changes such as endoplasmic ribosomes and Nissl bodies disintegration.^8^ Besides neurons, glial cells, including microglia and astrocytes, also experience morphological changes after ischemia. Ramified microglia can transform into an “activated state”, characterized by swollen ameboid-like cells, accompanied by the production of pro-inflammatory substances, including cytokines, chemokines, and reactive oxygen species (ROS),^9^ while astrocytes usually undergo gradual alterations both in molecular expression profiles and morphologies, which serves to protect neurons in the ischemic penumbra.^10,11^ After ischemia, increased blood-brain barrier (BBB) permeability contributes to the infiltration of several immune cells including leukocytes, monocytes, and macrophages, into the ischemic lesions, which release a variety of neurotoxic or neurotrophic factors to exert either neuroprotective or detrimental effects on ischemic brain tissues.^12–17^

**Fig. 1:**
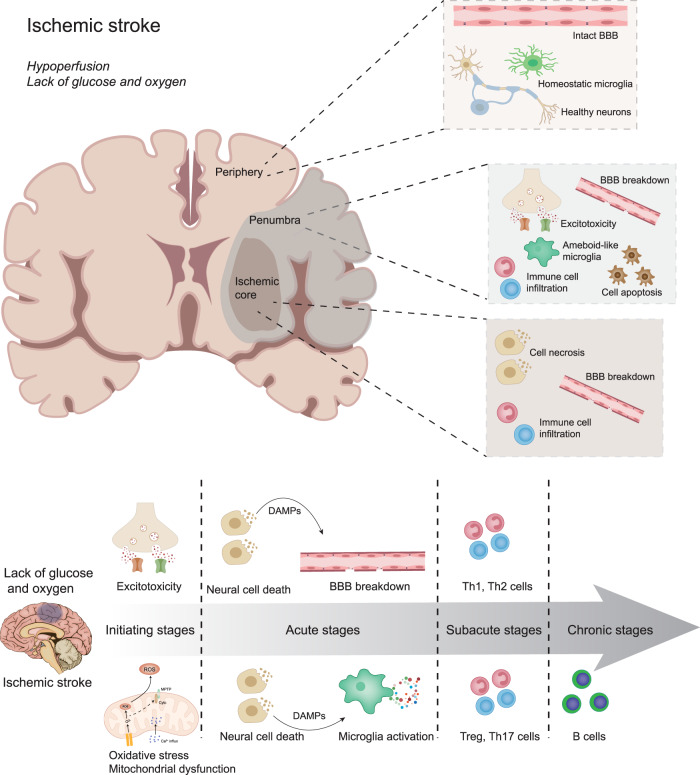
Spatial and temporal relationships of the pathophysiology in ischemic stroke. BBB Blood-brain barrier, DAMPs Damage-associated molecular patterns, Th1 T-helper cell 1, Th2 T helper cell 2

The temporal and spatial alterations in ischemic stroke are illustrated in Fig. 1.

### Experimental models of ischemic stroke

Efforts have been made to elucidate the pathophysiological mechanisms and screen potential therapeutical targets of ischemic stroke, and models both in vivo and in vitro are utilized to mimic ischemic circumstances. The most frequently used experimental ischemic stroke model is middle cerebral artery occlusion model (MCAO), in which a filament is utilized to block cerebral blood flow from the middle cerebral artery to induce a transient occlusion.^18,19^ This model was mostly used for studying blood-brain barrier disruption and inflammatory response in cerebral ischemia.^20,21^ Besides MCAO model, photothrombosis model is also utilized to induce cerebral ischemia in both mice and rats. In this model, Rose Bengal, a photosensitive dye is injected systematically into the animal, while a 532 nm wavelength laser is directly illuminated onto the skull and react with the photosensitive dye.^22^ Advantage of this model include the possibility to select a specific cortical brain region for ischemia and the high reproducibility with very low mortality.^18^ Correspondingly, the most frequently used in vitro model to mimic ischemic stroke is the oxygen and glucose deprivation (OGD) model, in which oxygen is replaced by N_2_ and glucose in the medium is omitted. Often this model is combined with cell co-cultures to study cellular interactions under ischemic circumstances.^23^ However, a limitation still remains that the in vitro model should be combined with in vivo studies to better comprehensively understand ischemic stroke.^18^

### Pathophysiological mechanisms involved in ischemic stroke

As a hallmark of ischemic stroke, interrupted cerebral blood flow depletes the brain of oxygen and glucose, which leads to disrupted ATP synthesis and energy deficiency, as well as impaired ion homeostasis and acid-base imbalance.^24,25^ All these dysfunctions result in cerebral neuropathological changes, such as brain edema, neuroinflammation, and neural cell death, eventually underpinning severe neurological deficits.^26^ Progress has been made in unveiling the pathogenesis and mechanisms of stroke, including cellular excitotoxicity,^27^ mitochondrial dysfunctions,^28^ neuroinflammation,^29^ BBB impairment,^30^ and cell death processes.^31^ Various signaling pathways become activated in these pathological transitions, and their targeted regulation could serve as a potential therapeutic strategy. Given the complex pathophysiology of ischemic stroke, the accompanying injury and signaling mechanisms should be first identified and then further elucidated to develop targeted interventions.

**Fig. 2:**
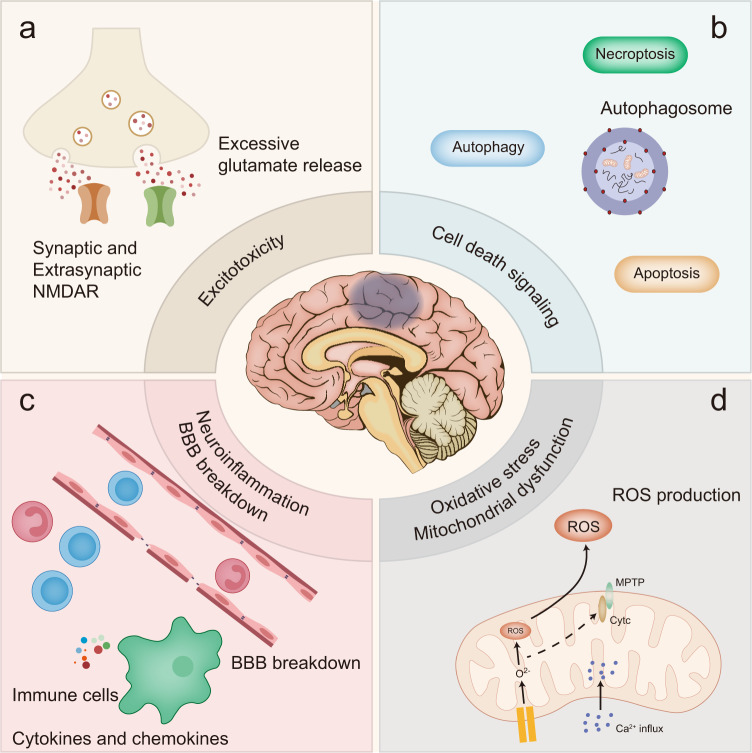
A brief summary for the pathophysiology involved in ischemic stroke. **a** Excitotoxicity in ischemic stroke, in which excessive glutamate are released and both synaptic and extra-synaptic NMDARs are involved; **b** Cell death signaling pathways, which mainly involves autophagy, apoptosis and necroptosis in ischemic stroke; **c** Neuroinflammation and BBB breakdown in ischemic stroke. Here we've presented the participation of various immune cells and chemokines and cytokines released, which thus contribute to blood-brain barrier breakdown; **d** Oxidative stress, which is mainly characterized by ROS production and mitochondrial dysfunction that involves Ca^2+^ influx into mitochondria and MPTP in ischemic stroke

The present review describes various signaling pathways associated with ischemic stroke pathophysiology (Fig. 2) and categorizes the corresponding therapeutic approaches (Table 1). Additionally, we summarize evidence from national clinical trials assessing therapies targeting ischemic stroke (Table 2).

**Table 1:** Therapies targeting the related signaling pathways involved in the corresponding pathophysiology in ischemic stroke

Drug/Therapy	Targeting signaling pathway	Major targeting pathophysiology	Authors	Citations	Applications
NA-1	GluN2B-PSD95-nNOS	Excitotoxicity	Chen et al.	^333^	Animals/Neuronal cultures
ZL006	GluN2B-PSD95-nNOS	Excitotoxicity	Zhou et al.	^426^	Mice/rat MCAO
IC87201	GluN2B-PSD95-nNOS	Excitotoxicity	Lai et al.; Bach et al.	^27,427^	In vitro
Tat-p53DM	DAPK1	Excitotoxicity	Pei et al.	^349^	Animals/Neuron in vitro
GluN2B^CT1292–1304^	DAPK1/ GluN2B-PSD95-nNOS	Excitotoxicity	McQueen et al.	^351^	Animals/Neuron in vitro
Tat-K13	PTEN	Excitotoxicity	Zhang et al.	^75^	Rat focal ischemia
Geniposide	GluN2A/AKT/ERK	Excitotoxicity	Huang et al.	^352^	Rat tMCAO
Electroacupuncture	PI3K/Akt	Excitotoxicity	Kim et al.	^428^	Animals/rat
Pseudoginsenoside-F11	Akt-Creb	Excitotoxicity	Liu et al.	^353^	Rat MCAO
TRPM2 (Gene knockout)	Akt/ERK	Excitotoxicity	Alim et al.	^354^	Mice MCAO
Tat-Panx308	Panx1	Excitotoxicity	Weilinger et al.	^429^	Mice/Rat brain slices
tBHQ	Nrf2/ARE	Oxidative stress	Hou et al.	^358^	Rat MCAO
Trans sodium crocetinate (TSC)	SIRT3/FOXO3a/SOD2	Oxidative stress	Chang et al.	^364^	Rat MCAO
Genipin	UCP2-SIRT3	Oxidative stress	Zhao et al.	^365^	Mice MCAO
CCL2/CCR2 gene knockout	CCL2/CCR2	Neuroinflammation	Wattananit et al.; Dimitrijevic et al.	^263,367^	Animals/mice MCAO
Resveratrol	TLR4/NF-Kb/STAT3	Neuroinflammation	Ghazavi et al.; Rahimifard et al.	^369,370^	Rat MCAO
Stevioside	TLR/NF-kB	Neuroinflammation	Zan et al.	^372^	Rat TBI
Progesterone	TLR4/NF-kB	Neuroinflammation	Hsieh et al.; Li et al.; Wang et al.	^373–375^	Rat MCAO
Tak242	TLR4	Neuroinflammation	Abdul et al.	^430^	Rat/In vitro
Isoquercetin	TLR4	Neuroinflammation	Shi et al.; Wang et al.	^431,432^	Animals/In vitro
Propofol	TLRs	Neuroinflammation	Gui et al.; Marik et al.	^433,434^	In vitro BV2/Review
Dexmedetomidine	HMGB1/TLR4/NF-kB	Neuroinflammation	Zhai et al.	^376^	Rat MCAO
XPro1595	TNFs	Neuroinflammation	Clausen et al.	^378^	Mice MCAO
cTfRMAb-TNFR	TNFs	Neuroinflammation	Zhou et al.; Sumbria et al.	^379,380^	Mice MCAO
Stnf-Αr1	TNFs	Neuroinflammation	Liguz-Lecznar et al.	^381^	Mice MCAO
Quercetin	Sirt	BBB	Yang et al.	^435^	Rat MCAO
Minocycline	Sirt3/proline hydroxylase-2/MMP	BBB	Yang et al.	^396^	HBMECs/Rat hypobaric hypoxia
Hydrogen sulfide	MMP9	BBB	Liu et al.	^397^	Mouse MCAO
Vagus nerve stimulation	MMP2/9	BBB	Yang et al.	^398^	Rat MCAO
Hyperbaric oxygen	MMP2	BBB	Michalski et al.	^399^	Rat MCAO
Metformin	AMPK	BBB	Liu et al.	^104^	Mice MCAO
OPC transplantation	Wnt-5a	BBB	Khan et al.	^400^	Rat MCAO
Patchouli alcohol	MAPK	BBB	Wei et al.	^436^	Mice MCAO
DL-3n-butylphthalide (NBP)	MAPK/AQP4/MMP9	BBB	Mamtilahun et a l.	^437^	Rat MCAO
FTY720	Akt	Autophagy	Hasegawa et al.; Wei et al.	^401,402^	Rat MCAO
Hydroxysafflor	Akt	Autophagy	Qi et al.	^438^	Rat MCAO
Selenium	PI3K/Mtor/Akt	Autophagy?	Yang et al.	^404^	Rat MCAO
Electroacupuncture	PI3K/Akt	Autophagy	Wang et al.	^439^	Rat MCAO
DHL	PI3K/Mtor/Akt	Autophagy	Meng et al.	^440^	In vitro OGD/R
Diosgenin	STAT2/HIKESHI	Autophagy	Zhang et al.	^441^	Rat MCAO
Stem cell-secreted vesicles	STAT3	Autophagy	Xia et al.	^411^	Rat MCAO/In vitro
Melatonin	PI3K-Akt	Autophagy	Yang et al.	^442^	Rat MCAO
MTMR14	PTEN	Autophagy	Pan et al.	^443^	Mice MCAO/In vitro
Sevoflurane	PTEN/AKT1/Mtorc1	Autophagy	Xue et al.	^444^	Rat MCAO
Remote ischemic preconditioning	PTEN/AKT1	Autophagy	Zhong et al.	^445^	Mice MCAO
Neuroprotectin D1	Wnt/β-catenin	Autophagy	Mu et al.	^446^	In vitro OGD/R
Electropuncture	Wnt	Autophagy	Chen et al.	^447^	MCAO Rat
SMXZF	AMPK-mTOR	Autophagy	Guo et al.; Wang et al.	^405,406^	Mice MCAO/In vitro
Puerarin	AMPK/Mtorc/ULK1	Autophagy	He et al.	^448^	Rat MCAO
Electroacupuncture	SIRT-FOXO1	Autophagy	Xu et al.	^413^	Rat MCAO
Luteolin	SIRT3/AMPK/Mtor	Autophagy	Liu et al.	^414^	Rat MCAO
Melatonin	SIRT1-BMAL1	Autophagy	Liu et al.	^415^	Mice MCAO
Proanthocyanidins	ERK	Apoptosis	Fu et al.	^449^	Mice MCAO
Beta-hydroxybutyrate	ERK/CREB/eNOS	Apoptosis	Li et al.	^416^	Rat MCAO/In vitro
MCC950	NLRP3	Apoptosis/Inflammasome	Ye et al.	^392^	Mice MCAO
Genistein	NLRP3	Apoptosis	Wang et al.	^393^	Mice MCAO
BML-275	AMPK	Apoptosis	Hu et al.	^420^	Mice MCAO
Glycine	AMPK/GSK-3β/HO-1	Apoptosis	Chen et al.	^421^	In vitro OGD/R
Apelin-13	AMPK	Apoptosis	Yang et al.	^450^	Mice MCAO
CTRP3	AMPK/SIRT1-PGC-1α	Apoptosis	Gao et al.	^451^	In vitro OGD/R
Rosuvastatin	Sirt1/NF-kB	Apoptosis	Yan et al.	^422^	Rat MCAO
Salvianolic acid B	SIRT1	Apoptosis	Lv et al.	^452^	Rat MCAO
Stem cell therapy	SIRT-NFkB	Apoptosis	Sarmah et al.	^453^	Rat MCAO
	MiRNA-29b/SIRT1/PGC-1	Apoptosis	Xu et al.	^424^	In vitro
Tetrahedral frameword nucleic acids	TLR2-MyD88-NF-kappa B	Apoptosis	Zhou et al.	^454^	Rat MCAO/In vitro
Chinese drugs	TLR4/MyD88/MAPK/NF-kappaB	Apoptosis	Gu et al.	^455^	Rat MCAO

**Table 2:** Clinical trials targeting the related signaling pathways involved in corresponding pathophysiology in ischemic stroke

Trial number	Trial name	Current Status	Study start and end date	Duration[y]	Phase	Sponsor	No.of participants	Type	Dose	Route	Time form stroke onset	Description (Signaling pathways)
NCT00591084	Safety and Pharmacokinetic Study of Carbamylated Erythropoietin (CEPO) to Treat Patients With Acute Ischemic Stroke	Compl	2005–2006	1	2	Hospital	199	ginsenoside-Rd	10 or 20 mg/d	IV	<72 h	Ca2+channel blocker
NCT00815763	Efficacy and Safety of Ginsenoside-Rd for Acute Ischemic Stroke	Compl	2006–2008	2	3	Hospital	390	ginsenoside-Rd	20 mg/d	IV	<72 h	Ca2+channel blocker
NCT02446977	Administration of CBG000592 (Riboflavin/Vitamin B2) in Patients With Acute Ischemic Stroke	Compl	2015–2015	1	2	Hospital	50	CBG000592	20 mg/d	IV	<3 h	FMN,FAD
NCT02930018	Safety and Efficacy of Nerinetide (NA-1) in Subjects Undergoing Endovascular Thrombectomy for Stroke	Compl	2017–2019	2	3	Industry	1105	NA-1	2.6 mg/kg	IV	<12 h	GluN2B-PSD95–nNOS interaction
NCT04462536	Efficacy and Safety of Nerinetide in Participants With Acute Ischemic Stroke Undergoing Endovascular Thrombectomy Excluding Thrombolysis	Recru	2020–2022	2	3	Industry	1020	NA-1	2.6 mg/kg	IV	<12 h	GluN2B-PSD95–nNOS interaction
NCT00728182	Evaluating Neuroprotection in Aneurysm Coiling Therapy	Compl	2008–2011	3	2	Industry	185	NA-1	2.6 mg/kg	IV	<72 h	GluN2B-PSD95–nNOS interaction
NCT02315443	Field Randomization of NA-1 Therapy in Early Responders	Recru	2015–2022	7	3	Industry	558	NA-1	2.6 mg/kg	IV	<3 h	GluN2B-PSD95–nNOS interaction
NCT02549846	AdminiStration of Statin On Acute Ischemic stRoke patienT Trial	Compl	2015–2017	2	4	University	270	Atorvastatin Pitavastain Rosuvastatin	20 mg/d 4 mg/d 5 mg/d	PO	<24 h	HMG-CoA Reductase Inhibitors
NCT04834388	Studying Anakinra to Reduce Secondary Brain Damage After Spontaneous Haemorrhagic Stroke	Not Recru	2021–2022	1	2	University Hospital	75	anakinra	100 or 500 mg	IV	<8 h	IL-1 system
NCT03737344	BLOC-ICH: Interleukin-1 Receptor Antagonist in Intracerebral Haemorrhage	Compl	2019–2021	2	2	University/College	25	IL-1Ra Kineret®	100 mg	SC	<8 h	IL-1 system
NCT02002390	Efficacy and Safety of FTY720 for Acute Stroke	Compl	2012–2014	2	2	Hospital	22	Fingolimod	0.5 mg	PO	<72 h	Inflammation
NCT04629872	Fingolimod in Endovascular Treatment of Ischemic Stroke	Recruiting	2020–2021	1	2	University Hospital	30	Fingolimod	0.5 mg	PO	<6–24 h	Inflammation
NCT04675762	Combinating Fingolimod With Alteplase Bridging With Thrombectomy in Acute Ischemic Stroke	Recruiting	2021–2022	1	2	University Hospital	118	Fingolimod	0.5 mg	PO	<24 h	Inflammation
NCT04419337	Pioglitazone and SGLT2 Inhibitors vs. DPP4 Inhibitors in Patients With Stroke	Recruiting	2021–2023	2	3	University Hospital	550	Metformin	/	PO	<3 months	Inflammation
NCT04069546	The Efficacy of Remote Ischemic Conditioning on Stroke-induced Immunodeficiency	Compl	2019–2020	1	Not applicable	University	46	Remote ischemic conditioning	Physical strategy	Physical strategy	<48 h	Inflammation
NCT00376207	Physical Activity After Stroke: How Does it Effect Chronical Inflammation and Insulin Sensitivity	Compl	2006–2007	1	Not applicable	Hospital	200	Physical exercise	/	Physical strategy	/	Inflammation
NCT02225834	Atorvastatin in Acute Stroke Treatment	Compl	2011–2014	3	4	University	50	Atorvastatin	80 mg	PO	<48 h	Inflammation
NCT00097318	Safety Study of Interferon Beta 1a to for Acute Stroke	Compl	2004–2011	7	1	NIH Clinical center	60	rh IFN-Beta 1a	11 mcg/22 mcg/44 mcg/66 mcg/88 mcg	PO	<24 h	Inflammation/BBB
NCT02878772	Vinpocetine Inhibits NF-κB-dependent Inflammation in Acute Ischemic Stroke Patients	Compl	2014–2015	1	2,3	University Hospital	60	Vinpocetine	30 mg	PO	<48 h	Inflammation/NF-Kb
NCT01831011	Mildronate for Acute Ischemic Stroke	Compl	2008–2010	2	2	Hospital	227	mildronate injection	500 mg/d	IV	<7 days	inhibitor of carnitine-dependent metabolism
NCT04479449	Efficacy and Safety of SP-8203 in Patients With Ischemic Stroke Requiring rtPA	Compl	2019–2020	1	2	Industry	178	SP-8203	80 mg/d	IV	<4.5 h	Matrix metalloprotease pathway
NCT02787278	Safety and Efficacy of Two Doses of SP-8203 in Patients With Ischemic Stroke Requiring rtPA	Compl	2016–2017	1	2a	Industry	80	SP-8203	80 or 160 mg/d	IV	<4.5 h	Matrix metalloprotease pathway
NCT00901381	Granulocyte-colony Stimulating Factor for Stem Cells Therapy for Acute Ischemic Stroke	Compl	2007–2009	2	2	Research Institute	20	Filgrastim	10 µg/kg	IH	<48 h	Multiple mechanisms(activation of endogenous bone marrow cells and neuroprotection)
NCT03394950	Butyphthalide in Combination With Recombinant Tissue Plasminogen Activator for Acute Ischemic Stroke	Compl	2018–2021	3	4	Hospital	120	Butyphthalide	25 mg	IV	<4.5 h	Multiple mechanisms(PMCA,SERCA)
NCT00796887	Randomized, Controlled Trial of Extended-Release Niacin (Niaspan®) to Augment Subacute Ischemic Stroke Recovery	Compl	2009–2012	3	2	Research Institute	28	Extended-Release Niacin	500 or 1000 mg/d	PO	3–7days	Multiple mechanisms(TNF-α,TGF-β,cAMP,HDL,LDL)
NCT03686163	Effects of Intranasal Nerve Growth Factor for Acute Ischemic Stroke	Compl	2016–2020	4	4	Hospital	106	Nerve Growth Factors	20 ug/d	IN	<72 h	Multiple mechanisms(TrkA)
NCT02828540	Clinical Trial to Assess the Efficacy and to Evaluate Safety of HT047 in Patients With Acute Ischemic Stroke	Compl	2016–2018	2	2	University	78	HT047	1500 or 2250 mg/d	PO	<14days	Multiple mechanisms (herbal extracts)
NCT01762163	Efficacy and Safety of Qizhitongluo Capsule in the Recovery Phase of Ischemic Stroke	Compl	2013–2016	3	4	University/College	622	Qizhitongluo Capsule Naoxintong Capsule	12 granules/d 12 granules/d	PO	15–28 days	Multiple mechanisms (traditional Chinese medicine)
NCT01958957	A Safety Study of Ginkgolides Meglumine Injection in the Treatment of Ischemic Stroke.	Compl	2013–2014	1	4	Industry	6300	Ginkgolides Meglumine Injection	25 mg/d	IV	0.5–6 months	Multiple mechanisms (traditional Chinese medicine)
NCT01919671	Tongxinluo Capsule in Ischemic Stroke Patients (TISS)	Compl	2014–2016	2	4	University Hospital	2007	Tongxinluo capsule	12 granules/d	PO	<72 h	Multiple mechanisms (traditional Chinese medicine, mainly vasodilator)
NCT04649398	Cerebral Nimodipine Concentrations Following Oral, Intra-venous, and Intra-arterial Administration	Recruiting	2020–2023	3	/	University	30	Nimodipine	60 mg	PO/IV	/	Neuroinflammation/BBB
NCT04734548	Phase Ib/IIa Clinical Study of ApTOLL for the Treatment of Acute Ischemic Stroke	Compl	2020–2022	2	1, 2	Ministry	151	ApTOLL	0.025 mg/kg–0.2 mg/kg	IV	<6 h	Neuroinflammation/TLR
NCT04453800	The Efficacy and Safety of Sofadil for Injection in the Treatment of Acute Ischemic Stroke	Compl	2016–2018	2	2	University Hospital	236	Sofadil	500, 750 or 1500 mg	IV	<6 h	NMDA
NCT04486430	Efficacy and Safety Study of Neu2000KWL for Acute Ischemic Stroke Patients Within 6 h of Onset	Compl	2017–2019	2	2	Hospital	236	Neu2000	500 mg/750 mg/1500 mg	IV	<6 h	NMDAR
NCT04453800	The Efficacy and Safety of Sofadil for Injection in the Treatment of Acute Ischemic Stroke	Compl	2016–2018	2	2	Hospital	236	Neu2000	500 mg/750 mg/1501 mg	IV	<6 h	NMDAR
NCT00059332	Field Administration of Stroke Therapy - Magnesium (FAST-MAG) Trial	Compl	2005–2013	8	3	Research Institute	1700	Magnesium sulfate	4 g	IV	<2 h	N-type Ca2+channel blocker
NCT01502761	Intra-arterial Magnesium Administration for Acute Stroke	Termi	2012–2016	4	1,2	University	4	Magnesium sulfate	0.75 or 1.5 g	IA		N-type Ca2+channel blocker
NCT02912663	Magnesium And Verapamil After Recanalization in Ischemia of the Cerebrum: a Clinical and Translational Study.	Compl	2017–2020	3	1	University	10	Magnesium sulfate Verapamil	1 g 10 mg	IA		N-type Ca2+channel blocker
NCT05032781	Intra-Arterial Neuroprotective Agents and Cold Saline in Ischemic Stroke Intervention	Recru	2021–2022	1	1	Industry	24	Magnesium sulfate Minocycline	2 or 4 g 2, 4 or 6 mg/kg	IA		N-type Ca2+channel blocker
NCT02505295	Selenium and Ischemic Stroke Outcome	Compl	2015–2018	3	Not applicable	University	44	Selenium	1000 mg	PO	<72 h	Oxidative stress
NCT03945526	Effect of Astaxanthin Supplementation on Plasma Malondialdehyde Levels and NIHSS of Stroke Patients	Compl	Mar, 2010-Jun, 2010	3 months(0.25)	1	University	24	Astaxanthine	2*8 mg	PO	<48 h	Oxidative stress
NCT03402204	Efficacy of High and Low-Dose Simvastatin on Vascular Oxidative Stress and Neurological Outcome in Patients With AIS	Compl	2014–2015	1	3	University	64	Simvastatin	10 mg/40 mg	PO	<24 h	Oxidative stress
NCT04931628	Efficacy and Safety of Salvianolic Acid on AIS	Not Recru	2022–2023	1	Not applicable	University Hospital	190	Salvianolic acid	100 mg	IV	<72 h	Oxidative stress
NCT03539445	Efficacy and Safety of Butylphthalide for Acute Ischemic Stroke Patients Receiving Intravenous Thrombolysis or Endovascular Treatment	Recruiting	2018–2022	4	3	Hospital	1200	Butylphthalide	0.2 g	IV	/	Oxidative stress
NCT02222714	Safety Evaluation of 3K3A-APC in Ischemic Stroke	Compl	2014–2017	3	2	Industry	110	3K3A-APC	120, 240, 360 or 540 ug/kg	IV	<4.5 h	PAR1
NCT04950920	Phase III Clinical Trial of Y-2 Sublingual Tablets in the Treatment of Acute Ischemic Stroke	Compl	2020–2022	2	/	University Hospital	900	Y-2 sublingual tablets	Edaravone 30 mg and d-borneol 6 mg	PO	<48 h	ROS/Neuroinflammation
NCT01949948	Study of Tenecteplase Versus Alteplase for Thrombolysis (Clot Dissolving) in Acute Ischemic Stroke	Compl	2012–2016	4	3	Hospital	1050	Tenecteplase	0.4 mg/kg	IV	<4.5 h	tPA
NCT01675115	Efficacy of BNG-1 to Treat Acute Ischemic Stroke	Compl	2012–2014	2	3	Hospital	129	BNG-1	9 g/d	PO	<10 days	Unknown
NCT01436487	Study to Examine the Effects of MultiStem in Ischemic Stroke	Compl	2011–2015	4	2	Industry	134	MultiStem	400 or 1200 million	IV	24–48 h	Unknown(Immunotherapy)
NCT02963376	A Phase Ib/II in Patients With Acute Ischemic Stroke	Compl	2017–2018	1	1	University	24	DDFPe	0.05, 0.10 or 0.17 ml/kg	IV	<12 h	Unknown(lactate)
NCT00756249	Safety Study of Carbamylated Erythropoietin (CEPO) to Treat Patients With Acute Ischemic Stroke	Compl	2007–2008	1	1	Industry	16	Lu AA24493	0.005, 0.05, 0.5, 5 or 50 mcg/kg	IV	12–48 h	Unknown(SHH /Patched/Smoothened,Mash1,frataxin)
NCT00870844	Safety and Pharmacokinetic Study of Carbamylated Erythropoietin (CEPO) to Treat Patients With Acute Ischemic Stroke	Compl	2009–2011	2	1	Industry	24	Lu AA24493	0.5, 5 or 50 mcg/kg	IV	<48 h	Unknown(SHH /Patched/Smoothened,Mash1,frataxin)
NCT01678534	Reparative Therapy in Acute Ischemic Stroke With Allogenic Mesenchymal Stem Cells From Adipose Tissue, Safety Assessment, a Randomised, Double Blind Placebo Controlled Single Center Pilot Clinical Trial (AMASCIS-01)	Compl	2014–2017	3	2	Hospital	19	Allogenic mesenchymal stem cells from adipose tissue	1 million units/kg	IV	12 h	Cell therapy
NCT01501773	Intravenous Autologous Bone Marrow-derived Stem Cells Therapy for Patients With Acute Ischemic Stroke	Compl	2008–2011	3	2	Industry	120	Autologous bone marrow stem cell	30–500 million	IV	Sudden onset	Cell therapy
NCT01845350	Safety of Autologous M2 Macrophage in Treatment of Non-Acute Stroke Patients	Compl	2013–2016	3	1	University	13	M2 macrophage introduction	Not applicalble	Intracathecal	3–12 months	Cell therapy
NCT01468064	Autologous Bone Marrow Stromal Cell and Endothelial Progenitor Cell Transplantation in Ischemic Stroke (AMETIS)	Compl	2011–2015	4	1/2	University	20	Genetic: Autologous BMSCs transplantation Genetic: Autologous EPCs transplantation Genetic: IV infusion of placebo	2.5 million cells per kg	IV	Within 7 days	Cell therapy

## Pathophysiology and signaling pathways involved in ischemic stroke

### Energy deficiency due to a lack of glucose and oxygen

Immediately after ischemic stroke, cerebral blood flow is significantly reduced, which limits the availability of glucose and oxygen, especially in neurons. Energy disruption leads to mitochondrial dysfunction and oxidative stress-induced damage, triggered by the production of ROS.^32^ Concurrently, energy deficiency contributes to an ionic imbalance that affects Na^+^, K^+^, and Ca^2+^ levels, leading to cell depolarization and prompting glutamate release.^33^ The excessive glutamate activates N-methyl-D-aspartate receptors (NMDARs), inducing toxicity, cell death, and finally severe damage of the central nervous system.^34–36^ Taken together, deficiency in glucose and oxygen may eventually lead to cellular excitotoxicity and mitochondrial dysfunctions, which serve as the initiating session of ischemia-induced damage and subsequently cause other cascade of injuries. In this section, the review focuses on the various signaling pathways involved in glutamate and NMDAR-induced cell toxicity, namely, excitotoxicity, as well as oxidative stress and mitochondrial dysfunction in ischemic stroke.

### Excitotoxicity and related signaling pathways

Glucose and oxygen deficiency during cerebral ischemia induces neuronal cell depolarization and glutamate release. The latter then stimulates Na^+^/Ca^2+^ channels coupled with NMDARs.^37^ Enhanced Ca^2+^ influx perturbs ionic homeostasis, resulting in Ca^2+^ overload in both the mitochondria^38–40^ and cytosol. These changes stimulate a variety of proteases, lipases, kinases, phosphatases, endonucleases, and free radicals,^41,42^ as well as biological processes causing cell death, such as calpain activation,^43^ oxidative stress, and mitochondrial impairment.^44,45^ Overall, these cellular dysfunctions are termed excitotoxicity and involve NMDARs, α-amino-3-hydroxy-5-methyl-4-isoxazolepropionic acid receptors, and kainite receptors.^1,46^

**Fig. 3:**
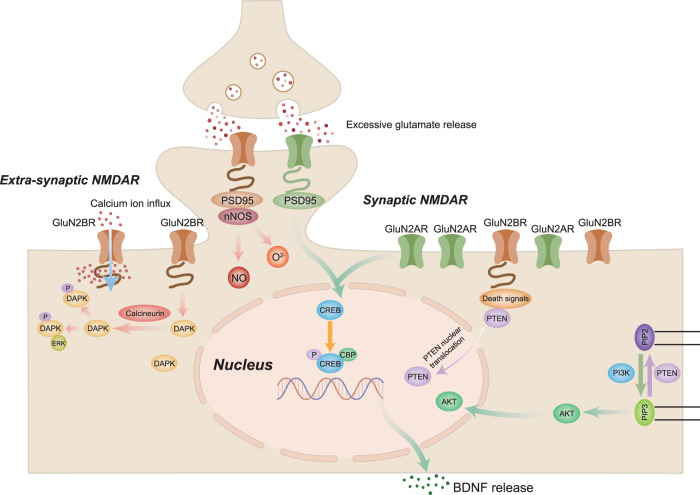
Excitotoxicity and signaling pathways involved in ischemic stroke. NMDAR N-methyl-D-aspartate receptors, PI3K Phosphatidylinositol 3 kinase, BDNF Brain-derived neurotrophic factor, CREB cAMP-response element-binding protein PTEN Phosphate and tension homology deleted on chromosome ten, PIP3 plasma membrane intrinsic protein 3, DAPK1 Death-associated protein kinase 1, PSD95 Postsynaptic density protein 95, nNOS Neuronal nitric oxide synthase

Despite their involvement in ischemic stroke-related excitotoxicity, NMDARs act as a double-edged sword. Functional and structural studies have revealed that activation of NMDARs containing the GluN2B subunit triggers excitotoxicity during ischemic stroke and subsequent neuronal apoptosis, whereas activation of NMDARs containing the GluN2A subunit exerts a neuroprotective effect.^33,47^ Similarly, it has been hypothesized that synaptic NMDARs promote neuronal survival, whereas extra-synaptic NMDARs play detrimental roles in neuronal activity.^48^ The analogy between synaptic vs. extra-synaptic NMDARs and GluN2A-containing NMDARs vs. GluN2B-containing NMDARs demonstrates the dual effect of NMDARs and their regulation of signaling pathways with neuroprotective or detrimental effects on ischemic stroke (Fig. 3).

#### Phosphatidylinositol 3-kinase (PI3K)-Akt signaling pathway

Stimulation of synaptic NMDARs activates the pro-survival PI3K/Akt signaling pathway, thereby exerting a neuroprotective effect. PI3K is an intracellular kinase classified into three categories (I, II, and III) based on structure and substrate specificity. In neurons, activation of the PI3K/Akt signaling pathway by NMDAR occurs via Ca^2+^ and calmodulin, which recruit phosphoinositide-dependent protein kinase 1. At the same time, Ca^2+^ triggers tyrosine phosphorylation of insulin receptor substrate 1, reinforcing NMDAR-induced Akt activation.^49–51^ The protective effect of the PI3K/Akt signaling pathway on ischemic stroke has been reported both in in vitro neurons during hypoxia^52–54^ and in vivo against ischemic neuronal death,^52,55–57^, and PI3K/Akt signaling inhibition aggravates ischemia-induced neuronal death in experimental stroke animals.^55,56,58,59^ Mechanistically, the neuroprotective effect of Akt is related to the phosphorylation and inactivation of various downstream targets, including glycogen synthase kinase 3 beta (GSK3β), pro-apoptotic B-cell lymphoma 2 (Bcl2)-associated BAD,^60^ c-Jun N-terminal kinase (JNK)/p38 activator ASK1,^61^ and apoptotic p53.^54^ These effects not only exist in neurons, but also in other neural cell types, which are possibly related to the inhibition of synaptic excitotoxicity and thus exert neuroprotective effects in ischemic stroke.

#### Brain-derived neurotrophic factor (BDNF) and cAMP-response element-binding protein (CREB)-related gene products

Synaptic NMDAR activation and Ca^2+^ influx activate the Ras/extracellular signal-regulated kinase (ERK) signaling pathway and nuclear Ca^2+^/calmodulin-dependent protein kinases, which in turn phosphorylate and activate CREB.^62,63^ Together with NMDAR and BDNF, CREB promotes the expression of numerous pro-neuronal survival genes.^64–67^ BDNF production in the brain relies on Ca^2+^ influx through NMDAR.^64,68,69^ Synaptic NMDARs promote BDNF gene expression,^70^ whereas extra-synaptic NMDARs block CREB-mediated BDNF expression.^71^ In experimental ischemic stroke models, BDNF is secreted into the brain and protects against ischemia-induced injury via neuronal GluN2A-NMDAR activation.^72,73^ Together, these results show that BDNF and, to some extent, the upstream CREB signaling pathway contribute to the neuroprotective effect associated with synaptic excitotoxicity in cerebral ischemia.

#### Phosphatase and tensin homolog (PTEN) signaling pathway

Extra-synaptic NMDARs are closely linked to signaling pathways associated with cell death and often contradict the effects triggered by synaptic NMDARs. Upon activation by Ca^2+^ influx through NMDARs, PTEN is recruited to GluN2B-NMDARs. The direct interaction between PTEN and the GluN1 subunit of GluN2B-NMDARs enhances current flow through the channel, tightening the junctions between PTEN and the neuronal death signaling complex. Concurrently, the excitotoxic stimulation of NMDARs initiates PTEN nuclear translocation, thus significantly lowering the phosphorylation of phosphatidylinositol-trisphosphate and Akt and consequently blocking PI3K/Akt signaling.^74,75^ Thus, contrary to the protective effect of PI3K/Akt, PTEN signaling may decrease cell survival and induce neuronal death.^76^ In agreement with this hypothesis, downregulating PTEN expression reportedly inhibits extra-synaptic NMDAR currents and protects neurons from experimental ischemic injury.^74^ The above evidence hints at the detrimental role of PTEN in ischemic stroke, which is largely mediated by regulation of extra-synaptic NMDAR activities.

#### Death-associated protein kinase 1 (DAPK1) signaling pathway

DAPK1 is a Ca^2+^/calmodulin-dependent serine/threonine-protein kinase, whose phosphorylation contributes to apoptotic cell death.^77,78^ DAPK1 participates in excitotoxicity in ischemic stroke. During ischemia, NMDAR overactivation promotes Ca^2+^ influx, activates Ca^2+^/calmodulin, and stimulates calcineurin phosphatase, which subsequently dephosphorylates and activates DAPK1.^79^ The latter is then transferred to the GluN2B subunit of NMDARs, aggravating ischemic injury.^80^ Preventing the interaction between GluN2B and DAPK1 attenuated neuronal excitotoxicity in mouse ischemic stroke models and downregulated the NMDAR current in vitro.^80^ In addition, NMDAR-regulated calcineurin activation contributes to DAPK1 activation, whereas NMDAR or calcineurin inhibition prevents DAPK1 dephosphorylation. DAPK1 inhibition protects against ischemic injury both *in cultured* neurons and in vivo, suggesting that potential treatments for ischemic stroke could be based on inhibiting DAPK1.^81^ It is interesting to note that the pro-survival signaling factor ERK serves as a downstream effector of DAPK1, and the DAPK1-ERK interaction could block the neuroprotective effect of ERK on experimental ischemic stroke, possibly by retaining ERK in neuronal cytoplasm.^82^

#### Postsynaptic density protein-95 (PSD95)/neuronal nitric oxide synthase (nNOS) signaling pathways and excitotoxicity-induced cell death

Neuronal NMDARs contribute to nitric oxide production, which is associated with calcium/calmodulin and is regulated by nNOS.^83^ NMDAR subunits bind directly to PSD95, which is composed of three PDZ domains.^84–86^ The binding of PSD95 to NMDAR and nNOS enhances Ca^2+^ influx, a hallmark of excitotoxicity.^87,88^ PSD95/nNOS signaling may play a pivotal role in ischemic stroke, as evidenced by the amelioration of neurological deficits in animals suffering from cerebral ischemia and whose nNOS activity was inhibited by either pharmacological or genetic means.^89^ Cerebral ischemia has been shown to enhance NMDAR/PSD95/nNOS interactions in neurons, thus further aggravating brain injuries after experimental ischemic stroke.^90^ All these results show that signaling through the PSD95/nNOS complex is crucial for excitotoxicity in ischemic stroke and contributes to the neurotoxic effects of extra-synaptic NMDARs.

### Mitochondrial dysfunction, oxidative stress, and related signaling pathways

**Fig. 4:**
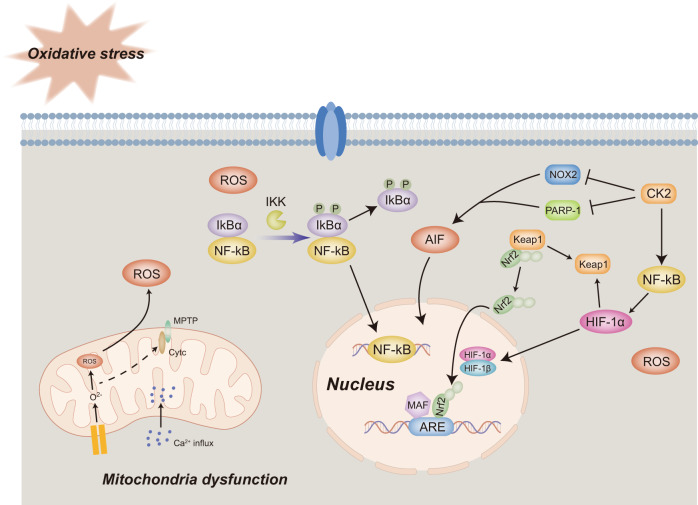
Oxidative stress and mitochondrial dysfunctions and signaling pathways involved in ischemic stroke. MPTP mitochondrial permeability transition pore, ROS Reactive oxygen species, ATP Adenosine triphosphate, HIF-1 Hypoxia-induced factor, Nrf2 Nuclear factor E2-related factor 2, ARE Antioxidant response element, CK2 Casein kinase 2, PARP-1 Poly ADP-ribose polymerase **1**, AIF Apoptosis-inducing factor, PINK1 PTEN induced putative kinase 1, NF-kB Necrosis factor-kB

Mitochondria are essential for maintaining energy homeostasis. When ATP synthesis and energy balance are disrupted by a lack of glucose and oxygen, the status and function of mitochondria become substantially altered. Ca^2+^ influx leads to mitochondrial permeability transition pore (MPTP) opening and cytochrome c release.^91,92^ At the same time, insufficient ATP supply triggers mitochondrial membrane depolarization, which is characterized by the influx of Na^+^ and efflux of K^+.^^93–95^ Besides mitochondrial dysfunction, energy deficiency in cerebral ischemia leads to oxidative stress, which severely damages cells and brain tissues.^96^ Oxidative stress accompanies several pathological processes and results from increased ROS production,^97^ mostly via oxidative phosphorylation in the mitochondria.^98^ Considering the intimate link between ROS and mitochondrial metabolism, mitochondrial dysfunction is often related to oxidative stress pathologies. During ischemia, oxidative damage and excessive Ca^2+^ levels contribute to MPTP induction, which further promotes succinate release and mitochondrial damage-associated molecular patterns including the activation of downstream inflammatory responses.^99–102^ Consequently, all these damaging factors lead to neurotoxic and cell death processes, in which a plethora of signaling pathways are involved (Fig. 4).

#### Hypoxia-inducible factor (HIF) signaling pathway

HIF-1, a key transcription factor activated during cerebral ischemia and hypoxia, comprises two subunits: HIF-1α and HIF-1β.^103–105^ HIF-1 enhances the expression of several glycolysis-associated genes under hypoxic conditions, thus helping cells and tissues become accustomed to hypoxia.^106^ Also, HIF-1α expression strongly correlates with ROS levels, with HIF-1α chains stabilized by the large quantities of ROS generated under hypoxia.^28,107^ In a positive feedback loop, lack of oxygen and glucose due to ischemia may enhance HIF-1 expression, thereby causing oxidative stress and further stimulating HIF-1 activity.

Conversely, it has been reported by other studies that HIF-1α may also play protective roles in the regulation of energy metabolism, especially in neurons. Consequently, HIF-1α depletion in mouse embryo fibroblasts results in excessive ROS, reduced glycolytic metabolism, and cell death.^108^ Besides controlling ROS production, the activation of HIF-1α may benefit cellular homeostasis by maintaining the redox equilibrium.^109^ Knockout of HIF-1α has been shown to disrupt redox homeostasis and glucose metabolism, such as pentose phosphate pathway and glucose transportation in SHSY5Y cell lines cultured under oxygen-glucose deprivation.^110^ In summary, HIF signaling may be closely associated with oxidative stress. Although there is still debate whether HIF-1α signaling enhances oxidative stress or not, activation of HIF-1α may be closely associated with production of ROS and oxidative stress, which consequently affects cellular redox equilibrium and biological activities.

#### Nuclear factor E2-related factor 2 (Nrf2) signaling pathway

Nrf2 regulates cellular redox homeostasis and counteracts oxidative stress. Nrf2 activation protects individuals against cerebral ischemic damage. In the resting state, Nrf2 is coupled to Keap1, its specific cytoplasmic receptor. The structure of Keap1 changes upon electrophilic or oxidative stress. As Nrf2 is phosphorylated through the protein kinase C pathway, it becomes uncoupled from Keap1, leading to enhanced expression of various anti-inflammatory proteins, antioxidant enzymes, and growth factors.^111,112^ In ischemic stroke, oxidative stress caused by elevated ROS levels induces Nrf2 accumulation in the nucleus, where it binds to antioxidant response elements (ARE) and maintains normal mitochondrial function.^113^ In contrast, insufficient Nrf2 contributes to neuronal mitochondrial depolarization, ATP depletion, and respiratory function impairment. suggested the beneficial role of Nrf2 in mitochondria.^114^

A variety of downstream signaling pathways, including PI3K/Akt, ERK/mitogen-activated protein kinase (MAPK), and nuclear factor kappa beta (NF-κB), potentially mediate the antioxidant effect of Nrf2 during ischemia. The neuroprotective PI3K/Akt pathway induces the nuclear translocation of Nrf2, which in turn stimulates the production of various antioxidants.^115,116^ Likewise, ERK/MAPK signaling pathway during ischemia is associated with a variety of neuroprotective biological processes, such as preventing apoptosis or enhancing Nrf2 phosphorylation and translocation.^117,118^ Also, NRF2 and NF-κB signaling pathways closely interact with each other under a variety of circumstances. On the one hand, deletion of NRF2 results in increased inflammation, as well as high levels of NF-κB; on the other hand, the elevated expression of NRF2 inhibits NF-κB-regulated pro-inflammatory and immune responses.^119^ This show the neuroprotective effects of NRF2 against NF-kB-induced inflammatory responses in cerebral ischemia.

In summary, Nrf2 is a crucial player against oxidative stress and mitochondrial dysfunction in ischemic brain injuries, possibly via the regulation of various downstream signaling pathways.

#### Casein kinase 2 (CK2) signaling pathway

CK2, an important oncogenic kinase, is crucial for counteracting ROS accumulation.^120^ First, it exerts a protective effect by inhibiting NADPH oxidase via regulating Rac1, a GTPase which significantly activate NADPH oxidase, possibly through interactions with other subunits and link the cytosolic subunits with the cell membrane.^121–123^ Second, CK2 reportedly phosphorylates Janus kinase and signal transducer and activator of transcription 3 (STAT3), enabling ROS detoxification by superoxide dismutase 2 (SOD2).^124,125^ Third, CK2 activates HIF-1α and phosphorylates NF-κB to promote the release of vascular endothelial growth factor (VEGF) and angiogenic proteins under in vitro hypoxic conditions.^126,127^ Conversely, CK2 inhibition in the ischemic region contributes to poly (ADP-ribose) polymerase 1 accumulation, which leads to the release of mitochondrial cytochrome c and apoptosis-inducing factor (AIF), with subsequent activation of downstream apoptotic events.^120^ These findings reveal the protective effect of CK2 against oxidative stress and inflammation, while promoting the release of angiogenic factors under hypoxia.

Notably, CK2 was shown to activate ROS-generating NADPH oxidase isoform 2 in an experimental ischemic stroke model, which induced AIF release into the mitochondria and subsequent DNA damage and apoptosis.^128^ Moreover, studies have shown that cyclin dependent kinase 5 and AKT/GSK3β are activated by CK2 in ischemia/reperfusion injuries.^129^ Given that inhibition of cyclin dependent kinase 5 reportedly alleviates cerebral ischemic stroke-induced damage, CK2 may do more harm than good.^130,131^ Taken together, the CK2 signaling pathway and related molecules play either protective or detrimental roles in ischemic stroke, especially in relation to oxidative stress. Importantly, downstream effectors of CK2 may function as potential targets against ischemic stroke.

#### Mitophagy and related signaling pathways

Mitophagy describes the process whereby mitochondrial content is taken up by mitochondria-derived vesicles and then transferred to lysosomes or peroxisomes for degradation. Mitophagy is essential for maintaining cellular homeostasis and serves as a protective strategy in various central nervous system diseases.^132^ Signaling pathways, such as PTEN induced kinase 1 (PINK1)/Parkin, Bcl2-interacting protein 3 (Bnip3), BNIP3-like, and FUN14 domain containing 1 pathways, are reportedly involved in mitophagy during ischemia–reperfusion. In the reperfusion stage, the levels of the free radical ONOO^−^ are increased, which leads to dynamin related protein 1 recruitment to the mitochondria and PINK1/Parkin-associated mitophagy.^133^ Meanwhile, elevated ROS levels upregulate the levels of Parkin RBR E3 ubiquitin protein ligase, which is recruited by PINK1, further exacerbating mitophagy.^134^ Interestingly, PINK1-regulated mitophagy is mechanistically associated with MPTP opening, whereas Bnip3-induced mitophagy is independent of MPTP.^135,136^

The activated mitophagy pathway may alleviate oxidative stress-induced cell injuries by promoting the degradation of damaged mitochondria.^137^ Enhanced mitophagy has been shown to possibly ameliorate ROS accumulation in cerebral ischemic stroke.^138^ In conclusion, mitophagy is significantly involved in the pathophysiology of ischemic stroke, along with the activation of various signaling pathways. Targeting these signals could potentially ameliorate the pathological changes and symptoms of ischemic stroke; however, the mechanisms remain to be elucidated.

### Cell death signaling pathways in ischemic stroke

**Fig. 5:**
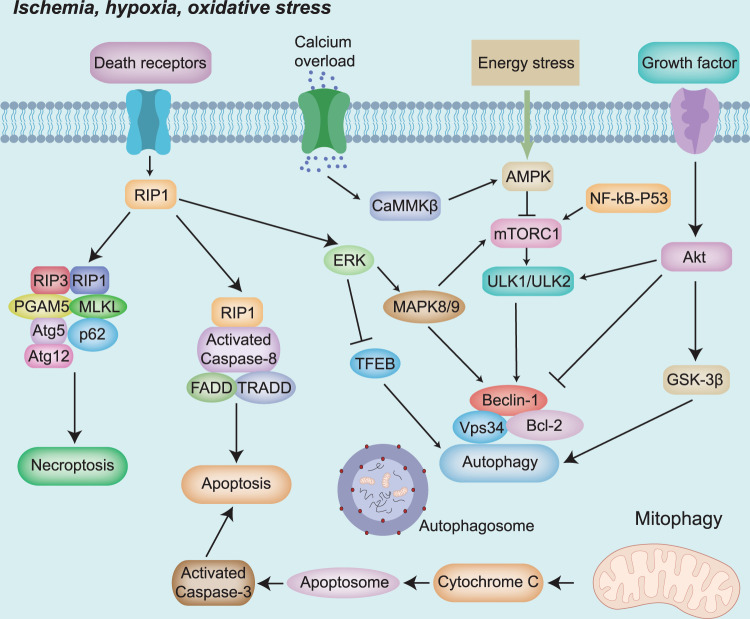
Cell death signaling pathways involved in ischemic stroke. GSK3β Glycogen synthase kinase-3β; Bcl-2 B-cell lymphoma-2; ERK Ras/extracellular signal-regulated kinase; CAMKs Ca^2+^/calmodulin-dependent protein kinases; MAPK Mitogen-activated protein kinase; TNF Tumor necrosis factor; mTOR mammalian target of rapamycin; AMPK 5′-AMP-activated protein kinase; FADD Fas-associating protein with a novel death domain; TRADD TNFRSF1A Associated Via Death Domain; RIPK Receptor-interacting protein kinase; MLKL Mixed lineage kinase domain-like protein; RIP1 Receptor interaction protein 1; RIP3 Receptor interaction protein 3; PGAM5 Phosphoglycerate Mutase Family Member 5; MLKL mixed lineage kinase domain like pseudokinase; Atg5 Autophagy related 5; Atg12 Autophagy related 12; TFEB Transcription factor EB; ULK1 Unc-51 Like Autophagy Activating Kinase 1; AMPK 5′-AMP-activated protein kinase; mTOR mammalian target of rapamycin; Apaf-1 Apoptotic peptidase activating factor 1

Damage caused by excitotoxicity, oxidative stress, and mitochondrial dysfunctions in ischemic stroke may induce a variety of cellular signaling cascades, which lead neural cells to undergo either programmed or unprogrammed death.^139^ Usually, programmed cell death includes apoptosis and autophagy, which are normal cellular functions,^140^ whereas unprogrammed cell death involves necrosis and is likely caused by external stimuli.^141^ Lack of oxygen and glucose in the ischemic core often leads to irreversible necrosis; in contrast, relatively minor damage in the penumbra is responsible for reversible death processes, such as apoptosis and autophagy.^40^ A variety of signaling pathways are highly involved in cell death, and they could either enhance or inhibit the process (Fig. 5).

### Signaling pathways related to autophagy in stroke

Autophagy is a self-protective pathway that maintains cell homeostasis and promotes cell survival by degrading circulating long-lived proteins, misfolded and aggregated proteins, and damaged organelles to obtain energy or in response to cellular stress.^142^ Subsequently, autolysosomes are newly formed to cleave the cargo for subsequent recycling.^143^ Emerging evidence indicates that autophagy is activated in various cell types following ischemic stroke, including neurons, glial cells, and endothelial cells. Autophagy can exert either beneficial or detrimental effects on cerebral ischemic injuries, as moderate autophagy may help degrade aggregated proteins,^144–146^ whereas inadequate or excessive autophagy may eventually lead to cell death.^147^ The dual role of autophagy in ischemic stroke may be explained by the involvement of multiple signaling pathways, such as mammalian target of rapamycin (mTOR), 5′-AMP-activated protein kinase (AMPK), MAPK, NF-κB, p53, HIF-1, and Bcl2 pathways.^148^

#### mTOR-related signaling pathways

mTOR is a serine/threonine protein kinase that comes in two major forms: mTORC1 (rapamycin-sensitive) and mTORC2 (rapamycin-insensitive). The former is responsible for cell growth and cell cycle progression, whereas the latter contributes to cellular skeleton formation. mTOR is a key regulator of the initial phase of autophagy, as it senses changes in signaling within the cell. Usually, mTOR limits autophagy by inhibiting phosphorylation of the Atg1/ULK1 protease complex.^149^ During ischemic stroke, mTOR interacts with multiple signaling pathway components that regulate autophagy,^150^ including PI3K/Akt, AMPK, and MAPK.

Akt, which is involved in various biological processes, can affect cellular autophagy through multiple signaling pathways, of which PI3K/Akt/mTOR is the most important one.^151–153^ The PI3K/Akt signaling pathway was suggested to exert a neuroprotective effect on ischemic stroke, possibly by regulating mTORC and, hence, autophagy in both mice MCAO models and OGD-treated primary neurons in vitro.^154^ Another study found that inhibition of mTOR by rapamycin activated the PI3K/Akt signaling pathway and, in turn, autophagy, thus protecting neonatal rats against hypoxia.^155^ Interestingly, homocysteine exerts a neurotoxic effect, possibly owing to excessive autophagy following downregulation of PI3K/Akt/mTOR signaling in neural stem cells, suggesting the bi-faceted role of autophagy in ischemic stroke.^156^

AMPK is a member of the serine/threonine kinase family and serves as an important endogenous defense factor against cerebral ischemia.^151^ During cerebral ischemia or hypoxia, energy deficiency and the consequent elevated AMP/ATP ratio contribute to AMPK phosphorylation, which activates autophagy to enhance energy production.^157^ Several studies on animal experimental ischemic stroke models have found that protective autophagy can be induced by regulating the AMPK/mTOR signaling pathway, thereby alleviating cerebral ischemic injury.^105,158^ A variety of downstream and upstream factors contribute to AMPK activity in both in vivo experimental ischemic stroke models and in vitro. Mechanistically, AMPK inhibits mTORC1 activity by phosphorylating and stimulating the TSC1/TSC2 complex during ischemia, thereby promoting autophagy.^24^ Furthermore, during ischemic stroke, Ca^2+^ overload can activate AMPK via calcium/calmodulin-dependent protein kinase beta and thus activate autophagy via the AMPK/mTOR pathway.^159^ Meanwhile, cytosolic p53 has been shown to directly inhibit autophagosome formation, whereas activated p53 functions to promote AMPKβ expression and inhibits mTOR expression to promote autophagy.^160^ These molecules contribute to the function of AMPK in autophagy in ischemic stroke.

MAPK is another important regulator of autophagy associated with ischemic stroke.^161^ MAPKs act as upstream regulators of mTORC1 and modulate autophagy through the MAPK/mTOR signaling pathway in ischemic stroke.^162^ Wang et al. found that autophagy protected against animal experimental cerebral ischemic injury through induction of an Akt-independent MAPK/mTOR signaling pathway, wherein ERK negatively regulated mTORC1.^163^ In contrast, Zhang et al. found that ERK negatively controlled autophagy by activating mTOR, which contributed to neuronal survival after experimental ischemic stroke injuries.^164^ Furthermore, an in vitro OGD/R study revealed that ERK could modulate autophagy by regulating mTOR in oxygen-glucose deprivation models.^165^ Therefore, the MAPK/ERK signaling pathway family could exert either a positive or negative regulation over mTOR in ischemic stroke; however, the exact mechanisms will require further investigation.

#### Beclin1/Bcl2 signaling pathway

Beclin1 plays a significant role in the early stage of autophagy. Local cerebral ischemia can upregulate Beclin1 expression and induce autophagy-like cell death, suggesting the involvement of Beclin1/Bcl2 signaling in the regulation of autophagy in ischemic stroke.^166^ Qi et al. found that Bcl2 phosphorylation after cerebral ischemia in rats perturbed the Beclin1-Bcl2 complex and triggered distal ischemic conditional autophagy, thereby alleviating mitochondrial damage.^167^ Moreover, peroxisome proliferator-activated receptor γ (PPAR-γ) expression increases during experimental cerebral ischemic injury. Activated PPAR-γ inhibits Beclin1-mediated autophagy, possibly by upregulating the expression of Bcl2/BclXL.^168^ Thus, either detrimental or neuroprotective factors impact on Beclin1-Bcl2 signal activities, subsequently affecting autophagy in ischemic stroke.

#### Other autophagy-related pathways

Several other signaling pathways are also involved in autophagy during ischemic stroke. Under ischemic conditions, the accumulation of misfolded proteins and disruption of Ca^2+^ homeostasis lead to self-protective events in the unfolded protein response (UPR) pathway.^151^ The UPR can promote autophagy by stimulating the PERK/eIF2 and Ire1/TRAF2/JNK pathways.^169^ The UPR signaling pathway mediator, activating transcription factor 6, can also affect autophagy in stroke.^105^ Rab7, a lysosome-associated small Rab GTPase, regulates autophagy during cerebral ischemia and provides neuroprotection against ischemic brain injury.^169^ Specifically, Rab7 enables the fusion of autophagosomes with lysosomes, thus affecting autophagosome maturation, lysosome formation, and maintenance of lysosomal function.^164^ However, the actual mechanisms of UPR signaling and that of Rab7 in ischemic stroke require further investigation.

### Signaling pathways related to apoptosis in stroke

Apoptosis is a highly regulated, energy-dependent form of cell death characterized by distinct morphological changes, such as cell shrinkage, cytoplasmic condensation, nuclear membrane breakdown, and apoptotic body formation.^170^ Apoptosis, especially neuronal apoptosis, is involved in the pathology of post-ischemic stroke. Cerebral ischemia leads to a decrease in ATP, which causes cellular apoptosis in the ischemic penumbra. Anti-apoptotic signals enable the potential recovery of dysfunctional neurons, while pro-apoptotic signals contribute to neuronal death, thus modulating the balance between pro-apoptotic and anti-apoptotic signals serve as potential therapeutic targets.^171^ Stroke triggers two principal apoptotic pathways: the extrinsic (or death receptor) pathway and the intrinsic (or mitochondrial) pathway. Initiated by a variety of both external and internal damaging stimuli, apoptosis eventually triggers a caspase cascade, which leads to the cellular injuries experienced during ischemic stroke.

#### Apoptosis by the extrinsic/death receptor pathway

The extrinsic apoptotic pathway is triggered by the combination of ligands, including TNF-α, FasL, and TRAIL, and the corresponding death receptors (TNF-α receptor 1, Fas/CD95/APO1, and TRAIL-R, respectively) on the cell surface.^172^ In the event of an ischemic stroke, the receptor recruits the death domain adaptor proteins FADD and TRADD, which form a complex by binding to procaspase-8.^173^ This complex induces a variety of downstream damaging processes and eventually leads to activation of caspase-8.^174^ Once activated, caspase-8 triggers downstream effector caspases, either directly via proteolytic cleavage or indirectly by cleaving BH3-interacting domain (BID) to its truncated form, which mediates apoptotic cell death via the mitochondria-dependent pathway.^175,176^ Furthermore, during ischemic injury, neurons and glial cells release TNF-α, increasing Fas mRNA and protein levels. These could function as stimuli for the extrinsic apoptotic pathway and ultimately lead to neuronal death.^31^

#### Apoptosis by the intrinsic/mitochondrial pathway

The intrinsic pathway, also called the mitochondrial pathway, is a receptor-independent signaling cascade that affects mitochondrial energy metabolism. Apoptotic stimuli, such as excessive Ca^2+^ accumulation and oxidative stress, mediate mitochondrial cell death.^177,178^ Lack of ATP due to oxygen and glucose deficiency results in cellular depolarization and excessive glutamate release, both of which further enhance Ca^2+^ influx.^179–183^ Ca^2+^ overload triggers calpain activation, which mediates the cleavage of Bcl2-interacting BID into its truncated active form, together with caspase-8 in the death receptor pathway.^177,184^ Truncated BID interacts with pro-apoptotic Bcl2 family members, forming a dimer and causing MPTP opening.^185^ These changes trigger the release of various pro-apoptotic factors, including cytochrome c, endonuclease G, and AIF,^186^ which ultimately lead to apoptosome formation by binding to apoptotic protease activating factor-1.^187^ Upon apoptosome formation, procaspase-9 becomes activated into caspase-9, which triggers downstream effector caspases (caspase-3, caspase-6, and caspase-7) that promote neuronal cell apoptosis.^31^

#### p53-mediated apoptotic pathway

Besides the extrinsic and intrinsic apoptotic pathways, another programmed cell death process activated by ischemic stroke depends primarily on p53. The tumor suppressor p53 becomes activated in ischemic areas of the brain, whereby it contributes to neuronal apoptosis. By translocating to the nucleus and binding to its specific DNA site, p53 induces apoptosis in ischemic brain cells.^188^ A plethora of detrimental signals could stimulate p53. One is DNA damage, which can activate the apoptotic pathway via p53 phosphorylation.^189^ Another is represented by hypoxia and oxidative stress, which can also upregulate p53 protein levels.^190^ Concurrently and mechanistically, some upstream cascade proteins, including JNKs, p38, DAPK, ASK1, and Notch may also lead to p53 activation.^31^ All these factors stimulate p53 activity and lead to cellular apoptosis in ischemic stroke.

p53-induced apoptosis involves a variety of downtream genes and molecules, such as the pro-apoptotic genes *Bax, Noxa, p53AIP1*, and *PUMA*, all of which act directly on mitochondria to induce apoptosis.^190^ Subsequently, p53 leads to the intrinsic apoptotic pathway, releasing pro-apoptotic factors, forming an apoptosome, activating effector caspases, and inducing neuronal apoptosis.^191^ In addition, p53 mediates apoptosis by inducing the expression of paternally expressed 3 and blocking cell survival signaling.^190^ All these processes contribute to the onset and progress of p53-mediated apoptosis.

#### Notch signaling pathways in apoptosis

Notch signaling pathways, the most important component of which is Notch1, plays pivotal roles in a variety of biological processes in the central nervous system. Activation of Notch1, as well as other signaling pathways, including NF-κB, p53, contributes to neuronal death processes. It has been reported that p53 and Pin1 are highly associated with Notch and NICD in ischemic stroke. As an important mediator of apoptosis, p53 is activated by damages such as hypoxia.^192^ The combination of Notch with p53 is crucial for neuronal apoptosis during ischemic stroke, which majorly involves stabilization of p53 and transcriptional regulation of p53 and NICD target genes.^193^ Besides, Pin1, an isomerase that regulates p53 transactivation, is deemed to be involved in the pathogenesis of ischemic stroke, which is also related with Notch signaling and is responsible for ischemic stroke-induced neuronal death and neurological deficits.^194^

In the meantime, studies have shown that Notch plays significant roles in modulating NF-κB-related cell death pathways. For instance, γ-secretase inhibitors down-regulate levels of NICD and protect against ischemic stroke damages. This protection effect is possibly via regulating NF-κB-related signals.^195^ Meanwhile, γ-secretase inhibitors block Notch signals and alleviates microglial activation.^196^ All these reveal the interactions between Notch and NF-kB pathways in both neurons and microglia in cerebral ischemia.

Besides, it has also been reported that ischemic stroke increases HIF-1α expression levels, which could directly bind with NICD and NF-κB.^197,198^ Inhibition of both γ-secretase/Notch and HIF-1α significantly reduced cell apoptosis, while enhanced expression of NICD and HIF-1α increased NF-kB levels. All these show the close interactions among NICD, p53, HIF-1α and NF-kB, which are highly associated with neuronal death processes, especially neuronal apoptosis in ischemic stroke.

#### Necrosis or necroptosis in cerebral ischemia

Following the onset of stroke, cerebral blood flow in the infarct area becomes significantly reduced, which induces necrotic death of resident neurons.^199^ Necrosis is an unprogrammed cell death process caused mainly by decreased ATP in ischemia.^31^ Recent studies have reported necrosis to be a highly regulated process involving various signaling pathways.^200^ The major downstream signaling pathways controlled by TNF-α include receptor-interacting protein kinase (RIPK1 and RIPK3) and mixed lineage kinase domain-like pathways.^201^

Facing cerebral ischemic damage, a complex containing TRADD, RIPK1, and ubiquitin 3 ligases is recruited by the combination of TNF-α and its TNFR1120 receptor. Complex IIb is subsequently activated in both ischemia and hypoxia, contributing to the phosphorylation and association of RIPK1 and RIPK3.^202–204^ Within the complex formed by this association, mixed lineage kinase domain-like is further activated by RIPK3, which eventually leads to cell death.^205^ Concurrently, a cascade of inflammatory reactions, including secretion of pro-inflammatory cytokines, favors necrosis damage and exacerbates ischemic brain injuries.^206^

#### Pyroptosis and ferroptosis in ischemic stroke

Majorly observed in ischemic penumbra, pyroptosis potentially induces pro-inflammatory pathways in ischemic stroke.^207^ During the process of pyroptosis, cells get swollen and cellular organelles are released to induce inflammation, in which caspase-1 is activated and form inflammasomes.^208^ All these contribute to pyroptotic cell death and secretion of inflammatory factors, such as IL-1β and IL-18.^208,209^

Another less mentioned but important cell death pathway is ferroptosis. Ferroptosis is regulated by peroxidation, which requires sufficient accessible iron.^210^ In ischemic brain regions, enhanced cellular excitotoxicity leads to the decrease in activity of GPX4 and reduction in GSH production,^211^ which accumulates excessive ferric ion and subsequently induces ferroptotic cell death. Also, damaged blood-brain barrier induces the iron to be transferred into neuronal cells, which further enhances ferroptosis.^212^ From another perspective, ferroptosis is also closely associated with oxidative stress, in which signaling pathways such as calcium-related signals, ATF4 and Keap1-Nrf2 signaling pathways play a role.^213^ Despite being less frequently discussed, ferroptosis may also be greatly involved in the pathogenesis of ischemic stroke,with a variety of signaling pathways potentially participating in.

### Neuroinflammation, BBB disintegration, and related signaling pathways in ischemic stroke

Inflammation is a key component of ischemic stroke pathologies. Existing in nearly all stages of ischemic stroke, neuroinflammation is initiated by the release of DAMPs from injured or dead cells. These DAMPs, including adenosine, heat shock proteins, high mobility group box 1, and interleukin-33, are subsequently recognized by corresponding immune cells, and then trigger a variety of downstream signaling pathways.^214,215^ During the whole process of inflammation, various immune cells including microglia, macrophages, and T lymphocytes are activated.^216,217^ Also, the production of inflammation-related cytokines are stimulated, as well as interferons or chemokines including monocyte chemoattractant protein-1 (MCP-1).^218^ Upregulation of levels of several adhesion molecules assists leukocytes in adhering to vascular surfaces,^219^ which facilitates the infiltration of immune cells. An abundance of pro-inflammatory cytokines leads to BBB disintegration via activation of endothelial cells and pericytes,^220,221^ along with the release of specific markers, such as von Willebrand factor and nerve growth factor.^222,223^ BBB leakage results in cerebral edema, as well as astrocytic aquaporin 4 expression.^224,225^ All these factors, including MCP-1, von Willebrand factor, nerve growth factor, and aquaporin4, could induce immune cell adhesion to the vascular wall and then infiltrate into the central nervous system, consequently contribute to BBB disintegration and cellular edema.

**Fig. 6:**
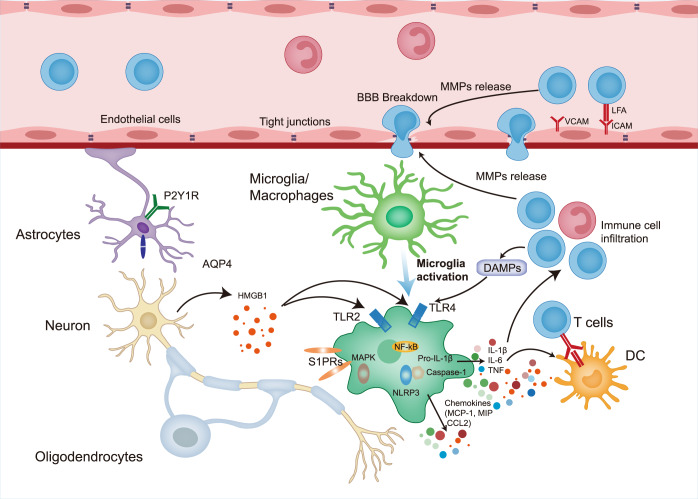
Neuroinflammation, BBB breakdown and related signaling pathways involved in ischemic stroke. DAMPs Damage-associated molecular patterns; AQP4 Aquaporin 4; HMGB1 High-mobility group box protein 1, TLR2 Toll-like receptor 2; TLR4 Toll-like receptor 4; MAPK Mitogen-activated protein kinase; NF-kB Necrosis factor-kB; NLRP3 Nod-like receptor protein-3; MCP-1 monocyte chemoattractant protein-1; MIP Macrophage inflammatory protein; CCL2 Chemokine-chemokine ligand 2; IL-1β Interleukin-1β; IL-6 Interleukin-6; TNF Tumor necrosis factor; BBB Blood-brain barrier; S1PRs Sphingosine-1-phosphate receptor; VCAM Vascular cell adhesion molecule; LFA Lymphocyte Function-associated Antigen; ICAM Intercellular cell adhesion molecule; DC Dendritic cells; MMP Matrix metalloproteinase

Several signaling pathways are involved in neuroinflammatory processes and BBB breakdown in ischemic stroke; they are strongly associated with each other and determine the pathophysiology of cerebral ischemia (Fig. 6).

### Cytokine- and chemokine-induced signaling pathways in neuroinflammation

During ischemic stroke, microglia, which represent the main resident immune cells in the brain, are the first cells recruited to infarct lesions. They secrete both pro-inflammatory cytokines, such as interleukin (IL)-1β, IL-6, and tumor necrosis factor alpha (TNF-α), and anti-inflammatory cytokines, including IL-1R antagonist (IL-1Ra) and IL-10.^15,226–228^ Together, these cytokines form a complex signaling network in ischemic stroke-induced neuroinflammation.

#### Cytokines

TNF is the most studied cytokine in ischemic stroke; it comprises a secreted form (solTNF) and a transmembrane form (tmTNF).^229^ The signal from both types of TNF is transferred via two different receptors, TNFR1 and TNFR2, respectively.^230^ The solTNF-TNFR1 signal is deemed responsible for pro-inflammatory effects of TNF, which trigger cell death signaling pathways. Instead, TNFR2 promotes cell growth and regeneration.^204,230,231^ Given the important regulatory role of TNF signals in inflammation and other neurological processes, TNFs are likely involved in the pathophysiology of ischemic stroke. Genome-wide association studies have identified a polymorphism in the TNF gene, which enhances stroke susceptibility, suggesting a pivotal role of TNF/TNFR1 in the etiopathogenesis of stroke.^232^ Moreover, TNF levels are significantly upregulated upon cerebral ischemia, whereby they mediate neuronal plasticity.^233^ As previously mentioned, TNF is secreted mainly by microglia, which protect against cerebral ischemia. Specific myeloid cell-TNF-knockout mice have been found to have larger infarct volumes and more severe neurological deficits than control mice.^227,234^ Removal of solTNF in mice reportedly alleviates the symptoms and pathology of cerebral ischemia, suggesting that elimination of solTNF and retention of tmTNF ameliorate cerebral ischemic injuries.^235^ Thus, different forms of TNF impact ischemic stroke, corroborating the important role of TNF in this disease.

The IL-1 family constitutes a huge and complex network of pleiotropic pro-inflammatory cytokines closely involved in regulating immune cells and inflammatory processes.^236^ Among IL-1 family members, IL-1α, IL-1β, and IL-1Rα have been studied in detail in relation to ischemic stroke. A polymorphism in the IL-1A gene has been associated with increased susceptibility to stroke;^237^ conversely, a polymorphism in the IL-1B gene lowers stroke risk.^238^ IL-1α expression is significantly increased in cerebral ischemia.^228^ Platelet-derived IL-1α contributes to neurovascular inflammation and causes the infiltration of neutrophils to ischemic lesions.^239^ Primarily secreted by microglia and macrophages,^15,240^ IL-1β affects neurons, glial cells, and the vasculature.^241^ IL-1β levels are significantly increased in the cerebrospinal fluid at days 2 and 3 post-stroke, suggesting a predictive value in stroke pathophysiology.^233,242^ The IL-1 family has been shown to exacerbate stroke pathology, as revealed by reduced infarct volumes in experimental ischemic stroke models of IL-1α/β knockout mice.^243^ Conversely, IL-1β administration worsens the outcomes of mice subjected to ischemic stroke.^244^ Overall, the IL-1 family plays a detrimental role in the pathophysiology of cerebral ischemic stroke and could serve as a potential therapeutic target.

Another vital member among pro-inflammatory interleukins is IL-6, which is secreted by a variety of cells, including monocytes, neurons, and glial cells.^245,246^ The IL-6 signaling pathways can be classified into classic signaling, which requires IL-6R and gp130, and trans-signaling, whereby IL-6 is linked to sIL-6R.^247^ Reportedly, the former is deemed to be neuroprotective and helps maintain neuronal homeostasis,^248^ whereas the latter contributes to IL-6-induced pro-inflammatory outcomes.^249,250^ IL-6 levels are upregulated during cerebral ischemia, which correlates with infarct volumes and survival rates.^251,252^ Interestingly, IL-6 levels are seemingly upregulated by IL-1β.^253^ The fact that brain-derived IL-6 promotes neurogenesis after stroke, and thus contributes to long-term functional recovery, points to its potential neuroprotective effect following cerebral ischemia.^254^ Even though only a few studies have focused on the role of IL-6 in ischemic stroke, its pleiotropic effects are worth further investigation.

Contrary to the aforementioned pro-inflammatory cytokines, IL-10 is released primarily by type-2 helper T cells and serves as an anti-inflammatory cytokine, reducing inflammation and limiting cellular apoptosis.^255^ IL-10 gene polymorphism is associated with the risk of stroke subtypes.^256^ In experimental ischemic stroke models, transgenic mice with enhanced IL-10 expression showed reduced infarct volumes and cellular apoptosis.^257^ Likewise, clinical studies have shown that low IL-10 levels correlate with poor stroke outcomes, worse neurological deficits, and extravagated inflammatory reactions.^258–260^ These results indicate that the anti-inflammatory properties of IL-10 serve as a potential clue for the diagnosis and prognosis of ischemic stroke.

#### Chemokines

In addition to cytokines, chemokines represent another group of small signaling proteins that contributes to the inflammatory processes in ischemic stroke. Immediately after cerebral ischemia, pro-inflammatory cytokines, such as TNF-α and IL-1β, induce the secretion of chemokines, such as MCP-1, fractalkine, macrophage inflammatory protein 1, microglial response factor-1, and cytokine-induced neutrophil chemoattractant.^261^ Chemokine-chemokine ligand 2 (CCL2) and its corresponding receptor, CCR2, are involved in regulating the inflammatory response in ischemia, possibly via immune cell recruitment and adhesion to cerebral endothelial cells.^151,262^ CCL2 expression becomes enhanced in the ischemic penumbra, cerebrospinal fluid, and serum after ischemia or ischemia-reperfusion.^153,263^ Moreover, CCL2/CCR2 expression correlates positively with infarct area and lesion enlargement,^151,262^ and enhanced CCL2 expression further aggravates ischemic injury in mice.^153^ Ischemic damage significantly increased MCP-1 mRNA (*CCL2*) expression, which further exacerbated ischemic brain injury, together with abundant infiltration of inflammatory cells in an experimental ischemic stroke model.^264^ All these findings suggest the detrimental role that CCL2/CCR2 signaling pathways play in ischemic stroke.

Besides the most frequently discussed CCL2, other chemokines are also involved in the pathogenesis of ischemic stroke. For instance, CCL3 has been reported to be upregulated in experimental ischemic stroke models.^265^ Consistently, external administration of CCL3 to brain ventricles exacerbated ischemia-induced injuries.^266^ Meanwhile, another chemokine CCL5 has been found to regulate ischemia/reperfusion (I/R) injuries in experimental ischemic stroke models.^267^ Clinical studies have also shown that plasma CCL5 levels were increased in symptomatic patients in comparison with asymptomatic ones.^268^ Besides the CC chemokine family, the CXC chemokines, also plays crucial roles in ischemic stroke pathogenesis. Among them, those ELR^+^ CXC chemokines, including CXCL1, CXCL2, and CXCL8, directly function to neutrophils toward ischemic brain regions; however, those ELR^−^ CXC chemokines, including CXCL10, CXCL12, and CXCL16, mainly induce Th1-cell infiltration in postischemic inflammation.^269^

### High-mobility group box protein 1 (HMGB1)/Toll-like receptor (TLR) and NF-κB signaling pathways in neuroinflammation

Various immune cells, as well as the corresponding cellular products, are associated with oxidative stress and necrosis activate the innate immune system, probably via the TLR signaling pathway. TLRs, which are expressed on both the cell surface and in the intracellular space, regulate the status and function of numerous immune cells.^270–275^ TLR signaling can be categorized based on two major downstream adaptor proteins: myeloid differentiation primary response 88 (MyD88)-dependent and adapter-inducing interferon-β-dependent pathways.^276^ Both TLR signaling pathways activate NF-κB, which subsequently triggers the release of pro-inflammatory cytokines.^277–279^

Interestingly, TLRs may act as another double-edged sword in ischemic stroke. In case of relatively moderate ischemic injury, TLR2 and TLR4/NF-κB signaling pathways are inhibited, whereas interferon regulatory factor 3 signaling is enhanced. Both of these processes exert neuroprotective effects on ischemia.^280^ Pretreatment with TLR2, TLR3, TLR4, TLR7, or TLR9 agonists alleviates the symptoms and pathological damage in various ischemic stroke models.^281,282^ Administration of lipopolysaccharide prior to ischemic insult protects against cerebral ischemia, possibly by modulating the TLR4 signaling pathway and inhibiting NF-κB after ischemic stroke attack.^283^ In contrast, elevated levels of plasma lipopolysaccharide appear to promote the expression of TLR4, causing the release of inflammatory cytokines, larger infarct volumes, and more severe functional deficits in rat cerebral ischemia models.^284^ These seemingly contradictory results suggest that LPS modulation of TLR4 response possibly depends on whether activation occurs before or after ischemic insult.

One key component in the TLR-related signaling pathway is HMGB1, which triggers downstream neuroinflammatory responses during stroke.^285^ HMGB1 levels are significantly elevated in the brain, specifically in microglia, astrocytes, and blood vessel cells, which are closely associated with neuroinflammation and cellular stress such as stroke.^286–289^ As one of the major ligands for TLRs, extracellular HMGB1 interacts with TLR2 or TLR4 and in turn NF-κB to elicit pro-inflammatory reactions.^280,290,291^ Moreover, the release of HMGB1 activates TLR4 and enhances IL-1β production through Nod-like receptor protein 3 (NLRP3) inflammasome activation.^292^ Furthermore, HMGB1 enhances the secretion of several pro-inflammatory cytokines, including inducible NOS, cytochrome c oxidase subunit 2, IL-1β, and TNF-α, promoting neuronal cell death during ischemia.^293^ These results suggest that both inflammatory reactions and cell death signaling pathways are induced by HMGB1/TLR signals in ischemic stroke, possibly aggravating ischemic injury.

### MAPK signaling pathway in inflammation and BBB dysfunction

MAPK comprises three main effectors: ERK1/2, JNK, and p38.^294^ Stress-activated protein kinases, JNK, p38 MAPK, and ERK exert detrimental effects during cerebral ischemia.^295^ Specifically, the MAPK signaling pathway is activated soon after the onset of ischemic injury, and p38 MAPK regulates the expression of various pro-inflammatory cytokines.^296^ Activation of the p38/MAPK/AR-related signaling pathway has been shown to promote the microglial pro-inflammatory phenotype in cerebral ischemia.^297^ Activation of MAPK/ERK signaling and consequent stimulation of metalloproteinase (MMP) expression could exacerbate BBB damage in ischemic stroke, further enhancing the expression of pro-inflammatory factors.^298^ Similarly, BBB damage in cerebral ischemia induced by a high-salt diet, has been associated with the p38/MAPK/SGK1 signaling pathway.^299^ These results suggest that MAPK-related signaling pathways exacerbate ischemic brain injury, possibly by enhancing neuroinflammatory processes and BBB dysfunction.

### MMPs and BBB dysfunction in ischemic stroke

MMPs are crucial for the function and structure of the BBB in both human and animal stroke models.^300,301^ The elevated production of MMPs and myeloperoxidase in ischemic stroke favors BBB breakdown.^302^ In particular, MMP9 induces proteolysis of the BBB basal lamina.^300,301^ Clinical studies have shown that baseline MMP9 serves as an important indicator of BBB disruption in ischemic stroke and is related to the hyperintense acute reperfusion injury marker used in magnetic resonance imaging.^303^ Hypothermia followed by rapid rewarming enhances the permeability of the BBB in ischemic stroke, along with elevated MMP9 expression levels and damage to tight junctions.^304^ MMP12 levels have been found to be elevated in rat cerebral ischemic stroke models, whereas suppressing MMP12 alleviates the symptoms induced by ischemia.^305^ Concurrently, MMP2 may participate in the pathophysiology of ischemic stroke, together with VEGF signaling. The latter is likely involved in the initial stages of ischemic stroke, whereby hypoxic preconditioning exacerbates BBB injury and brain edema.^306^ Furthermore, it has been shown that recovery from BBB damage is associated with both the MMP2 and VEGF pathways in acute cerebral ischemia, suggesting a close link between MMP2 and VEGF.^307^

### Sphingosine-1-phosphate receptor (S1PR)-related signaling pathways during neuroinflammation in ischemic stroke

S1PRs form a group of G protein-coupled receptors abundant in microglia and are thought to regulate inflammatory responses in ischemic stroke.^308^ In vitro studies have shown that the addition of S1P to microglia subjected to oxygen-glucose deprivation/reperfusion exacerbates hypoxia-induced neuronal apoptosis.^309^ In experimental ischemic stroke models, sphingosine kinase 1 phosphorylates sphingosine to S1P, which binds to S1PR3 and confers microglia a pro-inflammatory phenotype. Sphingosine kinase 1 enlarges the brain infarct volume and exacerbates neurological symptoms by upregulating the expression of pro-inflammatory cytokines.^310^ Intriguingly, the S1PR agonist fingolimod has been recently reported to switch microglia from a pro-inflammatory to an alternatively activated phenotype in a chronic hypo-perfused ischemic stroke model in mice.^311^ Thus, the pro-inflammatory mechanism of S1PRs in ischemic stroke requires further exploration.

### Inflammasome activation in ischemic stroke

Inflammasomes are large multiprotein complexes,^312,313^ which can mediate neuroinflammation and contribute to neural cell death in ischemic stroke.^314^ Both in vivo and in vitro model studies suggest that the NLRP3 inflammasome plays a pivotal role in microglia-associated neuroinflammation in ischemic stroke, possibly through alterations to the microglial phenotype.^315^ These effects may be linked to activation of the NF-κB signaling pathway.^316^ Additionally, NLRP1 is related to cerebral ischemic injuries, and its inhibition alleviates neuroinflammation in ischemia.^317^ Thus, inflammasome activation, either via NLRP1 or NLRP3, contributes to the pathogenesis of ischemic stroke and could provide a therapeutic target against cerebral ischemia.

### Microglial phagocytosis and complement activation

Microglia functions as the major phagocyte in the central nervous system, which is responsible for myelin debris clearance and pruning synapsis.^318^ It has been reported that microglia phagocytose tissue debris in experimental ischemic stroke model, which contribute to tissue repair and neuronal network reconstruction.^319,320^ However, other studies have also pointed out that over-enhanced microglia engulfment exacerbates cerebral ischemia-induced brain injuries.^321,322^ Hence, microglial phagocytosis may play both beneficial or detrimental roles in ischemic stroke. Microglia could engulf a variety of dying cells and debris, in which a plethora of signaling pathways are involved. TMEM16F is expressed by stressed neurons in ischemic stroke, which induces neurons to expose phospholipid phosphatidylserine (PS), an ‘eat-me’ signal. Consistently, knockdown of TMEM16F hindered microglial phagocytosing viable neurons in the penumbra after experimental ischemic stroke.^323^ Besides, triggering receptor expressed on myeloid cells (TREM2) signaling pathways are deemed to be greatly involved in microglial phagocytosis in ischemic stroke. TREM2 deficiency dampens microglial phagocytosis of neurons, which further exacerbates ischemic brain injuries,^319^ indicated the neuroprotective role of Trem2 in ischemic stroke.^324^

Another part of phagocytosis is the complement system, including C1q and C3. Upon activation, C3 is cleaved into C3a and C3b, of which C3b as well as its receptor, CR3, function together to regulate dying cells clearance.^325,326^ Meanwhile, C1q, the biggest component of the C1 complex, has been reported to strengthen microglial clearance of apoptotic cells in ischemic stroke.^327^ After ischemic stroke, microglial phagocytosis of both synapses and neurons was directed by activation of complement, which eventually contributes to cognitive decline.^328,329^ Thus, with a variety of signaling pathways involved, activation of the complement system may also be closely interacted with microglial phagocytosis, which possibly, greatly influence the pathologies of ischemic stroke.

### Therapeutic approaches targeting pathophysiological signaling pathways involved in ischemic stroke

So far, the only drug approved by FDA for treating ischemic stroke is tissue plasminogen activator (tPA), which breaks down the blood vessel clot.^8^ This therapy has several limitations, such as the therapeutic window is only 4.5 h, and treatment outside the therapeutic window could possibly result in cerebral hemorrhage.^330^ Progress have been made in discovering new therapeutic approaches against ischemic stroke. Current studies have shed lights on micro-RNA therapies, in which expression levels of miRNA are changed and apoptosis-related genes are subsequently mediated.^331^ Another potential treatment is cell therapy, which utilizes stem cells to differentiate.^332^ However, therapeutic approach is quite limited, and more research are need to discover new potential therapeutic strategy for ischemic stroke.

Given the pivotal roles the pathophysiology and signaling pathways play in ischemic stroke, numerous therapeutic approaches have been explored in both experimental and clinical studies, and several of them have been demonstrated to be effective in treatment of ischemic stroke (Table 2).

### Therapeutic approaches targeting excitotoxicity and related signaling pathways in cerebral ischemic stroke

#### Targeting the GluN2B-PSD95-nNOS complex

The GluN2B-PSD95-nNOS complex plays a central role in regulating NMDAR activity and related signaling pathways; therefore, it could potentially serve as a therapeutic target for cerebral ischemic stroke. The Tat-NR2B9c peptide, which binds to either PSD95 or nNOS, was shown to prevent downstream neurotoxic pathways and superoxide production.^333^ Furthermore, Tat-NR2B9c administration reportedly improved behavioral deficits, reduced infarct volumes, and retained the gene transcription profiles in animal ischemic stroke models.^334,335^ Another study reported that TAT-NR2B9c alleviated neuronal death and p38-induced damage in ischemic injury,^336^ while a clinical study found that it significantly decreased infarcts in ischemic stroke patients.^337^ Another small molecule called ZL006 has been found to disrupt the interaction between PSD95 and nNOS in ischemia, without affecting the normal functions of NMDARs and nNOS.^90,338^ Similarly, IC87201 has been found to disrupt pathogenic interactions between PSD95 and nNOS but without impairing normal nNOS activities.^27^ Finally, a study has shed light on Neu2000, a sulfasalazine derivative and GluN2B antagonist that selectively blocks NMDARs and scavenges free radicals, which exerted a neuroprotective effect in ischemic stroke.^339,340^ All this experimental evidence highlights the potential of treating ischemic stroke by targeting the GluN2B-PSD95-nNOS complex and preventing its participation in excitotoxicity. However, several shortcomings still exist. Although overactivation of NMDARs is acknowledged to be important in the etiology of cerebrovascular insults, the importance in physiological function has made the current NMDAR antagonists ‘undruggable’ for clinical application in ischemic stroke.^27,341^ Also, the therapeutic time window is relatively short, and safety issues including nausea, vomiting, cardiovascular and psychomimetic effects, remain to be considered.^342–348^

#### Targeting the DAPK1 signaling pathway

DAPK1 phosphorylates p53, a tumor suppressor that serves as one of its substrates. The interfering peptide Tat-p53DM^241–281^ inhibits specifically the downstream targets of DAPK1, such as the pro-apoptotic genes *Bax*, *Puma*, and *caspase-3*, which are also regulated by p53.^349^ The administration of Tat-p53DM^241–281^ was observed to significantly reduce infarct area and alleviate behavioral deficits in experimental ischemic stroke models.^350^ Another drug, GluN2B^CT1292–1304^, dissociates DAPK1 from the GluN2B subunit and protects neurons from ischemic injury.^351^ However, it still remains controversial that McQueen et al. have found that genetic depletion of DAPK1 could not alleviate excitotoxic and ischemic injuries in neurons.^351^ With possible uncertainties, these results indicate that DAPK1 inhibition could potentially alleviate ischemic brain damage through decreasing cellular excitotoxicity.

#### Targeting the PTEN-induced signaling pathway

Based on the function of PTEN in inhibiting the PI3K/Akt signaling pathway and inducing apoptotic cell death via excitotoxicity, regulating PTEN could possibly help ameliorate excitotoxicity and, in turn, neurological deficits in ischemic stroke. Genetic knockdown of PTEN was found to retain PI3K/Akt signaling while downregulating the extra-synaptic NMDAR current, which exerted a neuroprotective effect on an experimental ischemic stroke model.^74^ Pharmacologically, an interfering peptide, Tat-K13, was utilized to disrupt the cell death signaling pathway activated by PTEN.^75^ Tat-K13 exerted a neuroprotective effect in rats suffering from experimental ischemic stroke by reducing the size of the infarct lesion.^33,75^ These findings suggest that, owing to its link to PI3K/Akt signaling, the PTEN-related pathway could serve as a potential therapeutic target in the treatment of ischemic stroke.

#### Targeting the AKT signaling pathway

The iridoid glycoside geniposide has been reported to protect neurons from ischemic damage by activating the GluN2A/AKT/ERK signaling pathway.^352^ Accordingly, pseudoginsenoside-F11 prevents calpain1 activation while promoting the GluN2A-mediated AKT/CREB pathway.^353^ Genes involved in the modulation of NMDAR expression along the Akt/ERK pathway could also potentially serve as therapeutic targets. *TRPM2* knockout mice showed significantly smaller ischemic lesions, altered expression of GluN2A and GluN2B, and stimulation of pro-survival Akt and ERK signaling in an experimental ischemic stroke model.^354^ Overall, therapeutic approaches involving drugs, physical treatment, or gene modifications enhancing AKT-related signaling pathways and NMDAR activities could reinforce synaptic NMDAR activities and their neuroprotective effects in ischemic stroke.

#### Targeting the Panx1 signaling pathway

During ischemia, NMDAR activates Src kinases, which subsequently phosphorylate residue Y308 in the C-terminus of pannexin 1 (Panx1), leading to secondary ischemic currents.^355,356^ Preventing Panx1 phosphorylation may alleviate the symptoms and pathologies of ischemic stroke. Indeed, use of the interfering peptide Tat-Panx308 helped reduce infarct lesion size and alleviate sensorimotor deficit symptoms in middle cerebral artery occlusion (MCAO) rats, suggesting its effectiveness in treating ischemic stroke.^356^ In spite of the limited number of studies, regulation of Panx1 in excitotoxicity could represent a promising strategy for ischemic stroke treatment.

### Therapeutic approaches targeting signaling pathways to alleviate symptoms and damage caused by oxidative stress in ischemic stroke

#### Nrf2/ARE signaling pathway

The Nrf2/ARE signaling pathway contributes to the generation of numerous protective factors, such as anti-inflammatory proteins, antioxidant enzymes, and growth factors. Its antioxidant target genes include those encoding for heme oxygenase 1 (*HO1*), NADP(H) quinone dehydrogenase 1 (*NQO1*), and glutathione S-transferase (*GST*).^357^ Thus, regulation of the Nrf2/ARE signaling pathway could potentially protect against oxidative stress-induced damage in ischemic stroke. It has been reported that injection of tBHQ, an Nrf2 inducer, alleviates the symptoms of experimental cerebral ischemic stroke.^358^ Similarly, administration of metformin in cerebral ischemic stroke models alleviated oxidative stress-induced BBB damage, possibly through activation of the NRF2/ARE signaling pathway.^359^ In contrast, higher vulnerability and exacerbated brain damage were observed in cerebral ischemic stroke models of Nrf2-knockout mice.^360^ Generally, activating the Nrf2/ARE signaling pathway may confer a neuroprotective effect in cerebral ischemic stroke, which is associated with mitigation of oxidative stress.

#### Sirtuin (SIRT)/forkhead box O (FOXO) signaling pathway

SIRT1–7 play important roles in oxidative stress during ischemic stroke. The SIRT/FOXO signaling pathway has been shown to prevent oxidative stress in cerebral ischemia-reperfusion. SIRT1 exerts an antioxidant effect by activating either the FOXO family or PPAR-γ coactivator-1 and, as such, could serve as a potential therapeutic target.^361,362^ SIRT3 has been reported to enhance SOD2 activity and decrease ROS levels.^363^ Moreover, transsodium crocetinate protected animals from oxidative stress induced by cerebral ischemia–reperfusion injury, probably by activating the SIRT3/FOXO3a/SOD2 signaling pathway.^364^ Similarly, genipin was found to regulate the UCP2/SIRT3 signaling pathway and alleviate oxidative stress induced by cerebral ischemia.^365^ These findings reveal the potential of SIRT signaling pathways in therapeutic approaches against oxidative stress and ischemic stroke.

### Therapies targeting neuroinflammation-related signaling pathways

#### Chemokine-related signaling pathways

Therapeutic approaches regulating CCL2/CCR2 expression may alleviate the symptoms and pathologies of ischemic stroke. Whereas CCL2 gene disruption reduced infarct volume, CCR2 deletion reduced infarct size, while also improving locomotor ability of mice in an experimental ischemic stroke model.^263,366^ *CCR* knockout reduced infarct volumes and mortality of mice in experimental ischemic stroke models. However, it should be mentioned that hindering monocyte infiltration using an anti-CCR2 antibody delayed long-term behavioral recovery, along with decreased expression of anti-inflammatory genes in MCAO mice, suggesting a double-edged role of CCL2/CCR2 in ischemic stroke.^367^ Infarct size in rat MCAO models has been reduced also via inhibition of another chemokine, CCL23 (also known as MIP3α).^265^ Taken together, regulating chemokine expression, especially the CCL2/CCR2 signaling pathway, may serve as a potential therapeutic approach against cerebral ischemic stroke, although the harmful effects of such an intervention should be carefully considered.

#### TLR-associated signaling pathways

Considering the important role played by TLRs in neuroinflammation, several studies have demonstrated that TLR signaling could serve as a treatment target. Overexpression of miR-18a-5p downregulates the levels of TLR4 and TLR7, exerting a protective effect against ischemic injury in vitro.^368^ Resveratrol modulates microglial activity and improves ischemia-induced neurological symptoms by regulating the TLR4/NF-κB/STAT3 signaling pathway.^369–371^ Stevioside, a natural glycoside, protects against cerebral ischemia by inhibiting TLR/NF-κB pathway-mediated neuroinflammation.^372^ Moreover, treatment with progesterone and its metabolites has been shown to alleviate the symptoms of various cerebrovascular diseases by regulating the TLR4/NF-κB signaling pathway and inhibiting neuroinflammation.^373–375^ Similarly, dexmedetomidine has been proven effective against inflammatory reactions, oxidative stress, increased infarct volume, and brain edema in MCAO rats by inhibiting the HMGB1/TLR4/NF-κB signaling pathway.^376^ Interestingly, one study reported that activating TLR7 reduced infarct volume and neurological deficits by enhancing interferon expression.^377^ This observation is possibly associated with the dual effect of TLRs on neuroinflammation and ischemic stroke. In conclusion, regulation of TLR signaling has been revealed to attenuate neuroinflammation and, thus, protect against ischemic stroke. This therapeutic effect is possibly related to a variety of downstream molecules, including NF-κB and STAT3, whose modulation could promote the beneficial effects of TLRs in ischemic stroke.

#### Cytokine-related signaling pathways

Regulation of IL-1 and TNF cytokine families could also help attenuate ischemic stroke injuries. A study using a single intravenous dose of XPro1595 or etanercept, which targets TNFs, found that both compounds alleviated inflammatory reactions and enhanced locomotor abilities in a mouse model of focal cerebral ischemia; however, they did not decrease infarct volume.^378^ Another modified therapy, cTfRMAb-TNFR, which transfers TNFR across the BBB, has been reported to successfully reduce the infarct area and ameliorate neurological deficits.^379,380^ Similarly, a preclinical study demonstrated that sTNF-αR1 retained axonal plasticity in the cerebral cortex after stroke,^381^ which is in agreement with the results of another study showing that injection of solTNFR1 in dendritic cells alleviated infarct injury and inflammation after experimental stroke.^382^ However, it’s still worth mentioning that targeting both solTNF and tmTNF may concurrently raise the risk of cardiovascular and demyelinating disease.^383^ Due to the possible side effects of the anti-TNF therapies, more efforts should be made for more specific anti-TNF therapeutics.

IL-1Ra is the only therapeutic agent against IL-1-associated inflammation.^226^ Preclinical studies have shown that recombinant IL-1Ra protects against ischemia-induced injuries in rats^384,385^ and mice.^386^ Concomitantly, the first randomized, double-blind, placebo-controlled trial utilizing recombinant human IL-1Ra showed that patients receiving rhIL-1Ra displayed milder inflammatory reactions and nearly no disability 3 months after stroke.^387^ There’re several shortcomings that rhIL-1Ra crosses the BBB slowly and has relatively short half-life in the circulation to achieve effective and persistent therapeutic concentration.^388,389^ Also, there’re studies showing that IL-1Ra increased the possibility of poor mRS outcomes.^390^ Though that, IL-1Ra still has good perspectives in cerebral ischemic stroke treatment owing to its anti-inflammatory properties.

#### NLRP3 inflammasome

NLRP3 inflammasome regulation has been acknowledged as a potential therapeutic approach for ischemic stroke.^391^ Brilliant blue G, a P2X7R purinergic receptor antagonist, or MCC950, an NLRP3 inhibitor, not only attenuated cerebral infarct areas and neurological impairments but also inhibited caspase-3-associated neuronal apoptosis.^392^ Similarly, genistein, a natural phytoestrogen, has been reported to alleviate cerebral ischemia-induced injury in senescent mice by inhibiting NLRP3 inflammasome formation.^393^ An in vitro study revealed that treatment modulating the immunoproteasome/NF-κB/NLRP3 inflammasome signaling axis could work against hypoxia and ischemia, as well as prevent apoptosis.^394^ Therefore, inhibition of NLRP3 inflammasome formation could possibly attenuate ischemic stroke inflammatory processes and limit cell death.

### Therapeutic approaches targeting the BBB in ischemic stroke

#### Sirt signaling pathways

Protecting the BBB could help alleviate ischemic stroke. In an experimental rat model of stroke, hyperbaric oxygen treatment helped protect the BBB, potentially by regulating the ATP/NAD^+^/Sirt1 signaling pathway.^164^ Similarly, quercetin has been shown to protect the BBB and alleviate ischemia–reperfusion-induced injuries via activation of Sirt1 signals in rats.^395^ Minocycline has also been shown to ameliorate hypoxia-induced BBB disruption. This effect was mediated by the Sirt3/proline hydroxylase-2 degradation pathway, together with decreased levels of MMP2, MMP9, and VEGF, as well as upregulation of tight junction proteins.^396^

#### MMP inhibition for BBB protection

Given the indispensable role of MMPs, inhibition of the MMP signaling pathway may be beneficial in anti-stroke therapy. Administration of hydrogen sulfide donors may help ameliorate cerebral BBB damage, most likely via MMP9 inhibition.^397^ In addition, vagus nerve stimulation could help protect the BBB in ischemic damage by inhibiting MMP2/9-mediated tight junction protein disruption.^398^ Similarly, hyperbaric oxygen has been reported to stabilize the BBB in an experimental ischemic stroke model, possibly by blocking MMP2 activation.^399^ Finally, intra-arterial norcantharidin alleviated cerebral BBB damage by decreasing MMP9 expression in an experimental ischemic stroke model.^400^ These results suggest that regulation of MMP-related signaling pathways protects the BBB from ischemic stroke injuries.

### Cell death-related signaling pathways as targets for ischemic stroke treatment

#### Autophagy-related signaling pathways

A variety of signaling pathways related with autophagy, including Akt, AMPK, and others, has been shown to be potential therapeutic approaches against ischemic stroke. Fingolimod, a well-established sphingosine-1-phosphate receptor agonist, alleviates neurological deficits and reduces infarct areas by enhancing Akt signaling and ameliorating neuronal apoptosis,^401,402^ as well as regulating the mTOR/p70S6K autophagy signaling pathway in ischemic stroke models.^403^ Studies have also reported that selenium protects the BBB from ischemia-reperfusion injuries associated with PI3K/mTOR/AKT signaling pathway activation, which is possibly related to autophagy inhibition.^152,404^ As for the AMPK signaling pathway, SMXZF, a combination of Rb1, Rg1, schizandrin, and DT-13 (6:9:5:4), exerts a neuroprotective effect on cerebral ischemia-reperfusion injury, possibly by suppressing autophagy through regulation of the AMPK/mTOR and JNK signaling pathways, both in animals and oxygen-glucose deprivation/reperfusion models.^405,406^ Likewise, by activating AMPK-induced autophagy, ezetimibe ameliorates neuronal apoptosis and infarct volume, while improving neurological deficits in MCAO rat models.^407^ Finally, physical exercise induces AMPK activation and mTORC1 inhibition, thereby promoting autophagy, which consequently improves cerebral ischemia outcomes.^408–410^

Besides these two main target signals, additional autophagy-associated signaling pathways, related mainly to STAT, and SIRT, could also serve as targets for ischemic stroke therapies. Extracellular vesicles secreted by stem cells help mitigate ischemic brain damage, possibly by modulating STAT3-dependent autophagy, both in vivo and in vitro.^411^ In an experimental rat cerebral ischemia-reperfusion injury model, electroacupuncture mitigated neurological symptoms and related pathologies through inhibition of maladaptive autophagy and activation of the SIRT/FOXO1 signaling pathway.^412,413^ In addition, other signaling pathways involving SIRT, including SIRT3/AMPK/mTOR and SIRT1/BMAL1, are activated by luteolin and melatonin, respectively, and help protect against cerebral ischemia–reperfusion-induced injuries.^414,415^

#### Apoptosis-associated signaling pathways

Likewise, regulation of several signaling pathways, such as ERK/MAPK, AMPK and SIRT signaling pathways, are shown to mediate apoptosis in ischemic stroke. Beta-hydroxybutyrate ameliorates cerebral ischemic stroke injuries by suppressing apoptosis induced via oxidative stress and mitochondrial dysfunction, both in vivo and in vitro. The curative effects on apoptosis are probably associated with ERK/CREB/eNOS signaling pathway activation.^416^ Modulation of other ERK/MAPK signaling axes, including the MAPK/ERK/EGR1, CXCL13/ERK/MEK, and DAPK1/ERK signaling pathways, has also been shown to protect against ischemia-induced injuries both in vitro and in vivo.^417–419^ With respect to the AMPK signaling pathways, BML-275, an AMPK inhibitor, exerts a neuroprotective effect on cerebral ischemic stroke by downregulating cytochrome c and AIF expression, consequently blocking apoptosis.^420^ In addition, glycine was shown to attenuate cellular apoptosis and improve ischemic stroke damage by suppressing the AMPK/GSK3β/HO1 signaling pathway.^421^ SIRT signals are also possibly involved, as Rosuvastatin may exert protective effects on cerebral ischemia in rats through the Sirt1/NF-κB signaling pathway and inhibition of apoptosis.^422^ Stem cell therapies also attenuate ischemia-induced injuries, potentially through the SIRT/NF-κB signaling pathway.^423^ Finally, an in vitro study revealed that regulation of the miRNA-29b/SIRT1/PPAR-γ coactivator 1 alpha axis ameliorated oxygen-glucose deprivation-induced cell apoptosis, thus protecting cells from ischemia.^424^ All these reveal the potential of therapeutics against cellular apoptosis in ischemic stroke.

### National clinical trials of therapeutic approaches targeting ischemic stroke and signaling pathways

Clinical trials targeting the pathophysiology and the related signaling pathways mentioned above have been implemented with respect to ischemic stroke. For instance, the value of targeting cellular excitotoxicity in ischemic stroke has been recognized by investigators pursuing clinical trials with nerinetine (NA-1), the inhibitor of GluN2B-PSD95-nNOS complex (NCT02930018, NCT04462536, NCT00728182, NCT02315443), Neu2000 (NCT04486430), and sofadil (NCT04453800). In addition, several clinical trials focused on neuroinflammation during ischemic stroke have also been implemented, including those targeted IL-1 (NCT04834388, NCT03737344), S1P receptors (NCT02002390), and Toll-like receptors (TLRs) (NCT04734548). Furthermore, therapeutic approaches targeting oxidative stress in ischemic stroke have also been tested in clinical trials, such as selenium (NCT02505295), astaxanthine (NCT03945526), and simavastatin (NCT03402204). Concurrently, stem cell therapy is attracting much attention due to its potential for exerting significant therapeutic effects on stroke patients.^425^ Various types of cells, including allogenic mesenchymal stem cells from adipose tissue(NCT01678534), bone-marrow-derived stem cell (NCT01501773), endothelial progenitor cells (NCT01468064), and autologous M2 macrophages (NCT018453500) have been tested in clinical trials as a reparative therapy for acute ischemic stroke. All these reveal prospects for targeting the pathophysiology and related signaling pathways in treating ischemic stroke.

### Concluding remarks and future perspectives

Ischemic stroke is characterized by the blockade of cerebral blood flow caused by the presence of thrombi in the blood vessels and has an overwhelming effect on people’s health and their quality of life. In recent years, studies have sought to further elucidate the mechanisms of ischemic stroke. Nevertheless, the complex pathogenesis of ischemic stroke means that the participating signaling pathways need further comprehensive exploration. In this review, we summarized the signaling pathways involved in ischemic stroke and categorized them based on their specific pathophysiological roles in excitotoxicity, mitochondrial dysfunction, oxidative stress, neuroinflammation, and cell death. Because these signaling pathways are interconnected, combined therapeutic targets against ischemic stroke may be elucidated.

At present, recanalization of blood vessels via intravenous thrombolytic treatment or mechanical thrombectomy represents the major therapeutic approach for ischemic stroke. However, this is underscored by the lack of suitable pharmacological treatments, calling for the discovery of new therapeutic targets against ischemic stroke. In this review, we combed through existing therapeutic approaches and classified them according to their target signaling pathways. In conclusion, our review comprehensively elucidates the signaling pathways involved in the pathophysiology of ischemic stroke and also points out potential therapeutic approaches against ischemic stroke associated with those key signaling pathways.
