# Correction To: Signaling pathways involved in ischemic stroke: molecular mechanisms and therapeutic interventions

**DOI:** 10.1038/s41392-022-01129-1

**Published:** 2022-08-12

**Authors:** Chuan Qin, Sheng Yang, Yun-Hui Chu, Hang Zhang, Xiao-Wei Pang, Lian Chen, Luo-Qi Zhou, Man Chen, Dai-Shi Tian, Wei Wang

**Affiliations:** Department of Neurology, Tongji Hospital, Tongji Medical College, Huazhong University of Science and Technology, Wuhan, 430030 China

**Keywords:** Molecular neuroscience, Neurological disorders

Correction to: *Signal Transduction and Targeted Therapy* 10.1038/s41392-022-01064-1, published online 06 July 2022

After online publication of the article^1^ the authors noticed, the legend for Fig. 2 contains textual error and captions of Figs. 5 and 6 inadvertently swapped. The figure legends should have appeared as shown below.

**Fig. 2** A brief summary for the pathophysiology involved in ischemic stroke. **a** Excitotoxicity in ischemic stroke, in which excessive glutamate are released and both synaptic and extra-synaptic NMDARs are involved; **b** Cell death signaling pathways, which mainly involves autophagy, apoptosis and necroptosis in ischemic stroke; **c** Neuroinflammation and BBB breakdown in ischemic stroke. Here we’ve presented the participation of various immune cells and chemokines and cytokines released, which thus contribute to blood-brain barrier breakdown; **d** Oxidative stress, which is mainly characterized by ROS production and mitochondrial dysfunction that involves Ca2+ influx into mitochondria and MPTP in ischemic stroke

**Fig. 5** Cell death signaling pathways involved in ischemic stroke. GSK3β Glycogen synthase kinase-3β, Bcl-2 B-cell lymphoma-2, ERK Ras/extracellular signal-regulated kinase, CAMKs Ca2+/calmodulin-dependent protein kinases, MAPK Mitogen-activated protein kinase, TNF Tumor necrosis factor, mTOR mammalian target of rapamycin, AMPK 5′-AMP-activated protein kinase, FADD Fas-associating protein with a novel death domain, TRADD TNFRSF1A Associated Via Death Domain, RIPK Receptor-interacting protein kinase, MLKL Mixed lineage kinase domain-like protein, RIP1 Receptor interaction protein 1, RIP3 Receptor interaction protein 3, PGAM5 Phosphoglycerate Mutase Family Member 5, MLKL mixed lineage kinase domain like pseudokinase, Atg5 Autophagy related 5, Atg12 Autophagy related 12, TFEB Transcription factor EB, ULK1 Unc-51 Like Autophagy Activating Kinase 1, AMPK 5′-AMP-activated protein kinase, mTOR mammalian target of rapamycin, Apaf-1 Apoptotic peptidase activating factor 1

**Fig. 6** Neuroinflammation, BBB breakdown and related signaling pathways involved in ischemic stroke. DAMPs Damage-associated molecular patterns, AQP4 Aquaporin 4, HMGB1 High-mobility group box protein 1, TLR2 Toll-like receptor 2, TLR4 Toll-like receptor 4, MAPK Mitogen-activated protein kinase, NF-kB Necrosis factor-kB, NLRP3 Nod-like receptor protein-3, MCP-1 monocyte chemoattractant protein-1, MIP Macrophage inflammatory protein, CCL2 Chemokine-chemokine ligand 2, IL-1β Interleukin-1β, IL-6 Interleukin-6, TNF Tumor necrosis factor, BBB Blood-brain barrier, S1PRs Sphingosine-1-phosphate receptor, VCAM Vascular cell adhesion molecule, LFA Lymphocyte Function-associated Antigen, ICAM Intercellular cell adhesion molecule, DC Dendritic cells, MMP Matrix metalloproteinase

The original article has been corrected.
